# Habitat patches for newts in the face of climate change: local scale assessment combining niche modelling and graph theory

**DOI:** 10.1038/s41598-020-60479-4

**Published:** 2020-02-27

**Authors:** Clémentine Préau, Frédéric Grandjean, Yann Sellier, Miguel Gailledrat, Romain Bertrand, Francis Isselin-Nondedeu

**Affiliations:** 1 Réserve Naturelle Nationale du Pinail, GEREPI, Moulin de Chitré, 86210 Vouneuil-sur-Vienne, France; 2 Laboratoire Ecologie et Biologie des Interactions – UMR CNRS 7267 Equipe Ecologie Evolution Symbiose, Bâtiment B8-B35, 6, rue Michel Brunet, TSA 51106, 86073 Poitiers, Cedex France; 3 Département Aménagement et Environnement Ecole Polytechnique de l’Université de Tours, CNRS; UMR CNRS 7324 CITERES, 33-35 Allée Ferdinand de Lesseps, 37200 Tours, France; 4 Vienne Nature, 14 rue Jean Moulin, 86240 Fontaine-le-Comte, France; 5 Laboratoire Évolution & Diversité Biologique (EDB UMR 5174), IRD, CNRS, UPS, Université de Toulouse Midi-Pyrénées, Toulouse, France; 6 Institut Méditerranéen de Biodiversité et Ecologie, UMR CNRS-IRD, Avignon Université, Aix-Marseille Université, IUT d’Avignon, 337 chemin des Mainajariés, Site Agroparc PB 61207, 84911 Avignon, cedex 09 France

**Keywords:** Conservation biology, Biogeography

## Abstract

*Triturus cristatus* and *Triturus marmoratus* are two protected and declining newts occurring in the administrative department of Vienne, in France. They have limited dispersal abilities and rely on the connectivity between habitats and their suitability. In a warming climate, the locations of suitable habitats are expected to change, as is the connectivity. Here, we wondered how climate change might affect shifts in habitat suitability and connectivity of habitat patches, as connectivity is a key element enabling species to realize a potential range shift. We used ecological niche modelling (ENM), combining large-scale climate suitability with local scale, high-resolution habitat features, to identify suitable areas for the two species, under low and high warming scenarios (RCP 2.6 and RCP 8.5). We associated it with connectivity assessment through graph theory. The variable ‘small ponds’ contributed most to land cover-only ENMs for both species. Projections with climate change scenarios revealed a potential impact of warming on suitable habitat patches for newts, especially for *T. cristatus*. We observed a decrease in connectivity following a decrease in patch suitability. Our results highlight the important areas for newt habitat connectivity within the study area, and define those potentially threatened by climate warming. We provide information for prioritizing sites for acquisition, protection or restoration, and to advise landscape policies. Our framework is a useful and easily reproducible way to combine global climate requirements of the species with detailed information on species habitats and occurrence when available.

## Introduction

Amphibians are declining globally and have become a high-priority group in conservation^1^. The multiple threats on amphibians include loss and fragmentation of habitats, invasive species, diseases, human-induced pollution and climate change^2,3^. Climate change, through its effects on temperature and water availability, is likely to affect amphibian survival, phenology and distribution^4–6^. Ecological niche modelling (ENM) has facilitated the modelling of potential range shifts in amphibians at several scales, forecasting range expansion or reduction depending on species^7–9^. ENM can provide useful information with which to plan conservation actions in the face of climate and/or land use change^10,11^.

Whereas climate change is a driver of range shift at global^12^ and regional scales^13^, amphibian conservation at a local scale is closely related to the landscape mosaic, connectivity and degree of fragmentation^14,15^. Landscape connectivity is defined as “the degree to which the landscape facilitates or impedes movement among resource patches”^16^ and is a key parameter that should be taken into account in conservation planning^17^. Urban and Keitt^18^ advocated the study of landscape connectivity through graph theory, considering habitat patches as “nodes” connected by “edges”, which refer to ecological corridors. Landscape graphs can be used to represent ecological networks and to analyse connectivity between habitats, with, for instance, the purpose of prioritizing sites for protection, improving connectivity, or assessing potential effect of an urban development project^19,20^. In this approach, the landscape between habitat patches is represented by a “resistance” or “cost” which estimates the relationships between environmental variables and movement of the considered species^21^. Resistance surfaces can be categorical, with a cost associated with land cover types^22^; continuous, for example from an output of ENM, where resistance increases when habitat suitability decreases^23,24^; or binary, assuming that the influence of the matrix on species movement is homogenous between patches^25^. Ziółkowska *et al*.^26^ have compared the efficiency of such resistance surface representation on landscape connectivity assessment and they recommend using continuous resistance surface whenever possible. Using a habitat suitability map from ENM helps to overcome a lack of sufficient data. The use of ENM output is also a way to explore predictions of changes in high habitat suitability sites and the associated connectivity of species populations in the face of climate change^27^. In addition, combining connectivity analyses for a group of species allows accounting for the variability of ecological requirements and movements through the landscape matrix^28^.

Newts, like most amphibians, have both aquatic and terrestrial life stages. The maintenance of their populations depends on the presence of suitable habitats for wintering and breeding seasons, as well as on movements allowing migration to breeding sites and the dispersal of juveniles. Here, we expected climate warming to reduce the distribution of suitable habitats for two newt species, *Triturus marmoratus* and *Triturus cristatus*, within Vienne, an administrative department of the region Nouvelle-Aquitaine, in France. We also expected a decrease in the related connectivity of habitat patches, as it is a key element enabling species to track their climatic niche, especially for low mobility species such as newts. We benefited from high-resolution data on the presence of these two species, to produce detailed identification of suitable habitats for newts and assessment of the related connectivity. This study has the advantage of accounting for relevant landscape data that are often lacking at larger scales, such as small ponds^13^, and provides a valuable tool for environmental planning at the scale of an administrative department. We used ecological niche modelling, combining large-scale climate suitability with local scale, high-resolution habitat features to identify suitable habitat patches within a detailed map of the study area. We associated it with connectivity assessment through graph theory to identify key areas for maintaining connectivity for newts. Such a tool can help to define conservation action strategy.

## Materials and Methods

### Study area and studied species

The study area corresponded to the administrative department of Vienne (7,308 km²), in Nouvelle-Aquitaine, France. We divided the study area into 50 × 50 m cells to allow the inclusion of high resolution land cover data in local scale ENMs.

The great crested newt *Triturus cristatus* is a boreal species ranging from Western Europe (France, Great Britain) to eastern Russia^29^. The marbled newt *Triturus marmoratus* has a smaller range, covering the north of Spain and Portugal and western France^30^. Both species and their habitat are protected in France and Europe^31,32^, and classified as near threatened in national and regional red lists^33^ and as least concern at the global scale^29,30^. The two newt species co-occur in Vienne, *T. cristatus* being at the southern limit of its range, with some sites sheltering the hybrid *Triturus blasii* (*T. cristatus* × *T. marmoratus*).

### Modelling approach and connectivity analysis

#### Step 1: Global climate-only ENMs

Our method consisted of five steps (ESM 1). Steps 1, 2, 3 and 4 were repeated for each species. First, we ran climate-only ENMs across the full range of each species to account for their global climatic niche. For this first step, we used georeferenced data, gathered at a 10 × 10 km grid scale, across the full range of each species, from the Faune-France database (www.faune-france.org) and from the GBIF database^34,35^, compiling 8,599 points for *T. cristatus* and 3,708 points for *T. marmoratus* from 1970 to 2018. We used the Biomod2 platform for ensemble modelling^36^ under R software version 3.3.2, including bioclimatic variables from Worldclim, within a 10 × 10 km grid^37^. We applied a selection procedure proposed by Leroy *et al*.^38^ to select a set of uncorrelated variables based on Pearson correlation and variable importance for each species (see ESM 2 for variable description and correlation). Using this process we selected seven variables for *T. cristatus*: maximum temperature of warmest month, temperature annual range, mean temperature of wettest quarter, mean temperature of coldest quarter, annual precipitation, precipitation seasonality, and precipitation of driest quarter. We selected eight variables for *T. marmoratus*: annual mean temperature, mean diurnal range, temperature seasonality, mean temperature of wettest quarter, mean temperature of driest quarter, precipitation seasonality, precipitation of warmest quarter, and precipitation of coldest quarter. We ran the models with eight algorithms: grouping generalized additive model (GAM), generalized linear model (GLM), multivariate adaptative regression splines (MARS), artificial neural networks (ANN), flexible discriminant analysis (FDA), classification tree analysis (CTA), generalized boosting models (GBM) and random-forest (RF). We ran the models with five sets of 10,000 randomly selected pseudo-absences (PA), five runs per model. We adjusted weights of points to give equal weight for presence and PA. This process resulted in a total of 200 models. Model parameters are available in ESM 3. We split the observation dataset into 70% for training and 30% for evaluation of the area under the ROC curve (AUC-ROC^39^) and calculation of a True skill statistic (TSS^40^). We used ensemble modelling, including all runs with TSS over 0.7, to display central tendency across the modelling algorithms^41^. We assessed uncertainty in our ensemble models by calculating the coefficient of variation among single models used for ensemble modelling.

To increase the resolution of our outputs, we used variables at higher resolution to project our climate-only ENMs throughout Vienne^42–44^. According to Guisan *et al*.^45^, increasing resolution when projecting species distribution should be done cautiously, because the span of potential values across the range decreases when the resolution of the data decreases. Therefore, prior to projections, we verified that the range of values found for each selected variable within the calibration dataset actually contained the range of values of the corresponding variable within the projection dataset (see ESM 4 for values). In this way, we projected the ensemble models to current climate conditions at a 1 km² grid, using a dataset of maximum temperature, minimum temperature and precipitation, initially calculated for France, and different from Worldclim data (ESM 2). We also projected our ensemble models with data depicting future conditions calculated for France (see ESM 2 for description of datasets calculated for France for current and future conditions^13,46^). This allowed us to increase the spatial resolution of our results. We calculated the bioclimatic variables from these sets of data using the dismo package^47^. We verified a correlation between Worldclim bioclimatic variables and our set of variables under current conditions, allowing us to use the latter to project our models (see ESM 5 for correlation values between bioclimatic variables). We then forecasted the climate suitability for the species under future conditions accounting for two scenarios of climate change. The Representative Concentration Pathway (RCP) 2.6 scenario forecasts a peak in radiative forcing followed by a decline, to remain under 500 ppm by 2100, allowing to maintain the increase of global mean temperature below 2 °C^48^. The RCP 8.5 scenario forecasts a rise of radiative forcing up to more than 1,300 ppm by 2100, with no specific climate mitigation^48^. We assessed the transferability of our ensemble models to these datasets by calculating the dissimilarity between variables used for calibrating the models and variables used to project the models through multivariate environmental similarity surface (MESS^49^), for both current and future conditions, using the dismo package^47^. The MESS allows comparison of the values of the variables across the study area to the distribution of values at reference points (i.e. occurrence of the species), and thus identifies where extrapolation can be an issue. The MESS is similar to a BIOCLIM analysis, but it allows negative values. Negative values indicate locations where at least one variable has a value outside the range of environments displayed by the reference set^49^. The more the values are negative, the more the location displays a novel environment, and extrapolation of the model is needed^49^.

#### Step 2: Local land cover-only ENMs

For step 2, we ran local scale ENMs across the study area, with the same eight algorithms of Biomod2. Night surveys are organised every year in Vienne during the amphibian breeding season (February to May), by the naturalist association Vienne Nature (www.vienne-nature.fr/), as part of monitoring, inventorying and improving knowledge on amphibian distribution. In order to homogenize the prospecting effort, the department of Vienne was divided into a 10 × 10 km grid. For this step 2, we used georeferenced observations for the two newt species within the study area, collected by the volunteer and professional naturalists of Vienne Nature between 2000 and 2018, during these field surveys. The database compiled 187 points for the great crested newt *Triturus cristatus* and 435 points for the marbled newt *Triturus marmoratus*. We spatially reduced presence points of the species from a distance of 50 m around each point to minimize sampling bias and point clustering^50^, leaving 183 points for *T. cristatus* and 398 points for *T. marmoratus*.

We used a set of 15 land cover variables (see ESM 2 for variable description, source and original resolution) to account for land cover suitability for the species within the study area. Vienne Nature inventoried data on small ponds, large ponds and springs across Vienne through remote sensing, by combining mapped information and orthophotos (ESM 2). The use of this dataset for the study area did not allow investigation of land-use change scenarios in this study. We computed different proxies to define land cover variables. For some variables, namely coniferous forests, broadleaved forests, woody moorlands, hedges, crops, large ponds, beaches, dunes and sand, natural grasslands, pastures, orchards, and vineyards, we computed an index of surface-compactness (SC) in each cell of the 50 × 50 m grid, using ArcGIS 10.3. We chose this index because it accounts for both the area and the shape of the variable, and it is commonly used to characterize the shape of habitat patches in landscape connectivity assessments, particularly in urban planning. Compactness is a measure of shape, thus the broader the heart of a patch, the more it has potential to be a core habitat. The compactness = (4*Pi*surface of the patch)/(perimeter of the patch²)^51^, thus the measure of compactness increases with the width of the heart of the patch. The SC index is the product of the compactness and the area of a patch. For some other variables, namely water courses, small ponds, and springs, we computed the distance to the closest element in each cell. However, we kept the original definition of the variables ‘elevation’ and ‘urban areas’ (see ESM 2). We ran all models with the 15 variables and selected the most important ones for each species according to the method of Leroy *et al*.^38^ (see ESM 2 for variable correlation). In this way we selected five variables important for *T. cristatus*: small ponds, springs, elevation, broadleaved forests and water courses. We also selected five variables for *T. marmoratus*: small ponds, elevation, springs, broadleaved forests and crops.

We ran the models with ten sets of 1,000 pseudo-absences (PA) selected at a minimal distance of 50 m from our occurrences (disk method), as this method has shown to perform well with few presence data^52^. We used five runs per model. We adjusted weights of points to give equal weight for presence and PA. This resulted in a total of 400 models. Model parameters are available in ESM 3. We used ensemble models to forecast the land cover suitability of the species under current conditions and displayed the related coefficient of variation between single models. We assessed the extent of extrapolation of the ensemble model through MESS^49^. We used 70% of the data for training and 30% for the evaluation of models by AUC-ROC and TSS.

#### Step 3: Habitat suitability index

At step 3, we calculated a map of habitat suitability index (HSI) accounting for both climate-only ENMs and land cover-only ENMs outputs from steps 1 and 2. The outputs of climate-only ENMs were continuous probabilities within cells of 1 × 1 km and the outputs of land cover-only ENMs were continuous probabilities within cells of 50 × 50 m. To combine the outputs of both types of ENMs, we disaggregated the outputs of climate-only ENMs at 50 × 50 m, keeping the same values as in larger original cells. We multiplied the outputs of climate-only and land cover-only ENMs for current conditions to obtain a HSI map varying from 0 to 1 for current conditions^53,54^. We multiplied the outputs of climate-only ENMs for future conditions and land cover-only ENMs for current conditions to obtain HSI maps for future conditions. This framework implies two underlying assumptions, which may lead to uncertainty: multiplying probabilities implies that climate and land cover have independent effects on habitat suitability, and multiplying current land cover-only ENM outputs with outputs of climate-only ENMs for future conditions implies no change of land cover in the future. We computed a global Moran’s I index on the residuals of current HSI maps^55^ to assess the spatial autocorrelation at species locations. This index ranges from −1 to 1, respectively standing for strong negative and strong positive spatial autocorrelation. A value of 0 indicates a random pattern.

#### Step 4: Landscape graphs and connectivity analysis

At step 4, we transformed the HSI map into a binary map using a threshold defined by the 10th percentile of training presence points^56,57^. This threshold allows removal of locations where HSI is lower than the suitability values of the bottom 10% of species occurrence, which could be affected by errors in the data or represent sink populations^56^. Locations with HSI lower than the threshold are considered as unsuitable whereas locations with HSI equal or higher than the threshold are considered as suitable. We used Graphab 2.2.6 to compute the landscape graphs and the connectivity analysis^58^. The nodes of the graphs corresponded to the patches of suitable habitat identified in the binary map of habitat suitability. We averaged the value of HSI across the cells composing habitat patches to obtain a value of capacity for each habitat patch. The capacity is considered as an indicator of the demographic potential of a patch. The value of capacity attributed to each habitat patch was equal to the mean of the HSI within the cells of each habitat patch, thus a value between 0 and 1. We calculated a resistance surface from the continuous HSI resulting from step 3, as: resistance = 1 when HSI was ≥ threshold; and resistance = e(ln(0.001)/threshold × HSI) × 10³ when HSI was <threshold^23,59^. The resulting resistance surface ranges from 1 to 1000. The edges were calculated in cost distance from the resistance surfaces. The graph allows one to network the suitable habitat patches (i.e. nodes), connected by the least costly edge between patches.

Once the graph was computed, we calculated a measure of landscape connectivity on the graph. We used the local metric of interaction flux (IF) to quantify the potential connectivity at the level of habitat patches. IF is the sum of the products of one habitat patch capacity relative to those of all other habitat patches, weighted by their probability of interaction, and it ranges from 0 to the sum of capacities^19,28^. IF represents the local contribution of each habitat patch to the global connectivity within the study area^28^. The calculation of the IF involves specifying a distance relative to dispersal of the species. While dispersal in pond breeding amphibians is mostly achieved by juveniles^60^, most studies on amphibians are conducted on adult individuals, where radio tracking is easier, and movements attributed to newts are in the order of a few hundred meters^28,61,62^. Based on our field experience, we decided to use a maximum dispersal distance of 1 km for both newt species. We used the interpolation tool of Graphab to compute a spatial generalization of the IF in every pixel of the study area, using a decreasing weighting function from the edge of the patches^28,63^. By assuming that individuals can be found outside habitat patches^64^, interpolation allows assigning a value of the patch-based connectivity metric IF outside the patches considered as suitable to the species and thus the assessment of the potential connectivity of any pixel within the study area. This value decreases when distance to the patches increases^28^.

#### Step 5: Two-species combination and assessment of potential changes

Finally, at step 5, we combined the interpolations of the IF for each species in a two-species map by averaging the single maps of IF values computed for the two species at step 4^28^. Because values of the IF depends on different graphs, we normalized the results by the mean and standard deviation, prior to combination, following the method of Sahraoui *et al*.^28^, to obtain comparable values for the two species, for current and future conditions. We evaluated the potential effect of climate change on overall connectivity for newts by computing the difference between two-species connectivity for current and future conditions.

## Results

### Global climate-only and local land cover-only ENMs

**Figure 1:**
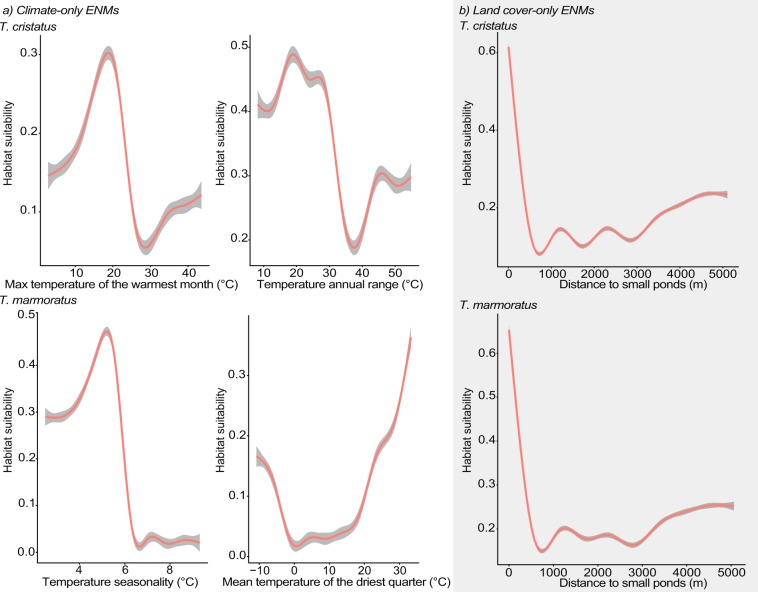
Response curves of the most important variables from (**a**) Climate-only ENMs and (**b**) Land cover-only ENMs for *T. cristatus* and *T. marmoratus*. Confidence interval is represented by grey background behind response curves.

For global climate-only ENMs, we respectively obtained a value of TSS and AUC of 0.788 and 0.964 for the ensemble model of *T. cristatus* and 0.921 and 0.985 for the ensemble model of *T. marmoratus*. We found little variation (±0 to 0.1) between models combined in the final climate-only ensemble models (ESM 6). We did not find dissimilarity between values used to calibrate the models and values used to project the models in current conditions (0 to 50; see ESM 7 for MESS analysis in current conditions). For *T. marmoratus*, we did not find dissimilarity for projections for 2050 and 2100 under RCP 2.6 nor for 2050 under RCP 8.5, and we found little dissimilarity leading to little extrapolation in projection for 2100 under RCP 8.5 (−50 to 0; see ESM 7 for MESS analysis for *T. marmoratus* in future conditions). However, for *T. cristatus*, we found high dissimilarity leading to high extrapolation in all projections with future scenarios (−8000 to −4000; see ESM 7 for MESS analysis for *T. cristatus* in future conditions). Because of the high dissimilarity between current and future conditions, the results presented for *T. cristatus* in future conditions should be treated with caution. For *T. cristatus*, maximum temperature of the warmest month and temperature annual range showed the highest variable importance, with the highest habitat suitability around 20 °C for both variables (Fig. 1a). For *T. marmoratus* the most important variables were temperature seasonality, with highest habitat suitability around 5 °C, and mean temperature of the driest quarter, with highest habitat suitability around 30 °C (ESM 8, Fig. 1a).

For local land-cover-only ENMs, we obtained a value for TSS and AUC of 0.769 and 0.96 respectively for the ensemble model of *T. cristatus*, and 0.687 and 0.926 respectively for the ensemble model of *T. marmoratus*. We found very little variation between models (±0 to 0.2) in the suitable areas identified in final ensemble models for both species (ESM 6). We found dissimilarity leading to little extrapolation for the land cover-only ENMs, especially in some areas in the northern quarter of the study area for both species and in the southeast for *T. cristatus* (−163 to 0; ESM 7). Distance to small ponds showed high variable importance compared to other land cover variables for both species (ESM 8); habitat suitability dropped when distance to small pond increased (Fig. 1b).

### Expected change in habitat suitability index

After combining climate-only and land cover-only ENMs, we found about 15% of the study area to be suitable for *T. cristatus* and about 24% to be suitable for *T. marmoratus* (Table 1, ESM 9). With climate change scenarios, we forecasted a decrease to less than 5% of the study area for *T. cristatus* under RCP 2.6 and we did not find any cells suitable for *T. cristatus* under RCP 8.5. The decrease in suitable areas was less important for *T. marmoratus*, for which suitable cells were mostly conserved under RCP 2.6. However, under RCP 8.5, the suitability of the study area decreased to about 20% in 2050, then was reduced to zero in 2100, as for *T. cristatus*. The suitable habitats of the two species overlapped in 10% of the study area in current climate conditions. The overlap was reduced to about 2% under RCP 2.6 and to zero under RCP 8.5. We did not find any significant spatial auto-correlation in models whatever the species considered (Moran’s I index = 0.0797 for *T. cristatus* (p-value = 0.118) and Moran’s I index = 0.008 for *T. marmoratus* (p-value = 0.078) (ESM 10)).

**Table 1:** Area of total suitable habitat in km² (climate-only ENM output × land cover-only ENM output) for the two species within the study area, for current conditions and scenarios of future conditions.

	*T. cristatus*	*T. marmoratus*
Current conditions	1120.09	1730.99
2050 RCP 2.6	151.32	1713.14
2100 RCP 2.6	187.36	1729.74
2050 RCP 8.5	0	1443.53
2100 RCP 8.5	0	0

### Landscape graphs and connectivity analysis

**Figure 2:**
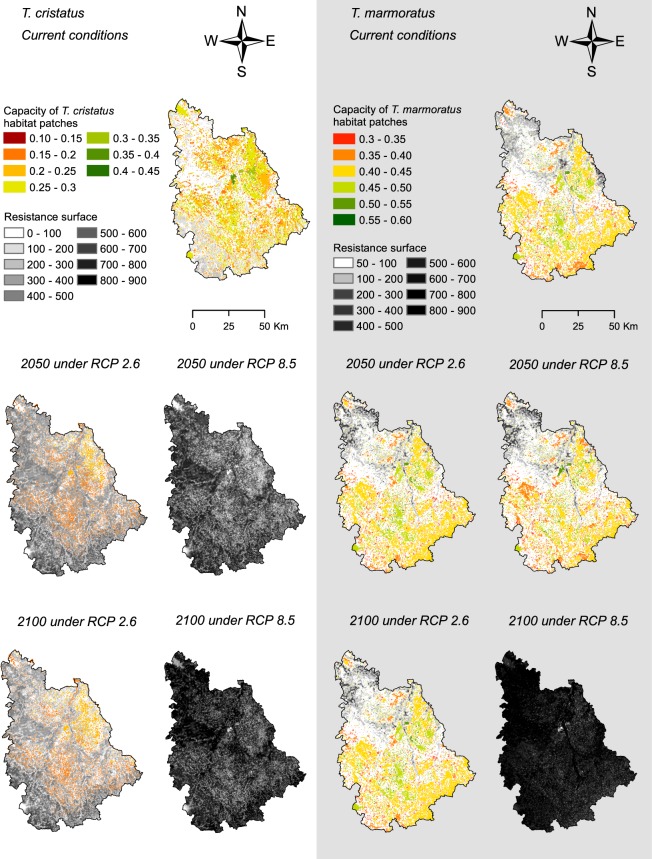
Habitat patches and resistance surfaces resulting from habitat suitability maps of *T. cristatus* and *T. marmoratus*, for use in landscape graphs computation. Habitat patches correspond to the nodes and resistance surfaces are used to compute the edges in the graph. Capacity is the mean value of HSI within a habitat patch.

In current conditions, the northeast quarter of the study area seemed to hold some of the most suitable habitat patches (i.e. with highest capacity) for the two newt species, in addition to those of the centre-east of the study area for *T. marmoratus* (Fig. 2). The southwest quarter of the study area seemed less suitable for both species, but there were generally more habitat patches for *T. marmoratus* than for *T. cristatus* in the south of the study area. As the resistance of the landscape increased, the number and capacity of habitat patches decreased under RCP 2.6 for *T. cristatus* (Fig. 2) and we did not find any habitat patch suitable for *T. cristatus* under RCP 8.5. For *T. marmoratus*, although the resistance of the landscape increased under RCP 2.6 and in 2050 under RCP 8.5, the habitat patches seemed more sustainable for this species than for *T. cristatus* (Fig. 2). However, the high resistance of the landscape and low suitability did not allow any remaining habitat patches for *T. marmoratus* in 2100 under RCP 8.5. The high resistance of the landscape and the lack of suitable habitat patches for 2050 and 2100 for *T. cristatus* and for 2100 for *T. marmoratus* when we considered the scenario RCP 8.5 did not allow us to compute landscape graphs under such conditions.

**Figure 3:**
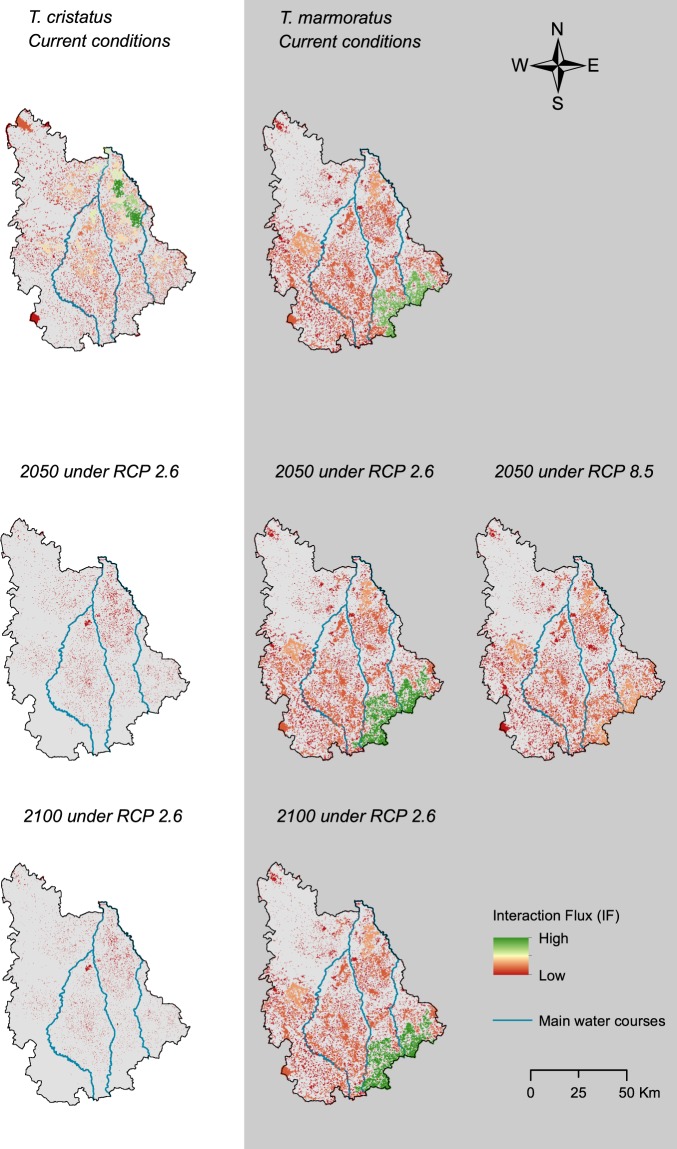
Contribution of habitat patches to global connectivity through the study area identified through the interaction flux (IF, see materials and methods section for explanation) for *T. cristatus* and *T. marmoratus*.

The calculation of the IF metric at the level of habitat patches showed that the most connected habitat patches were located in the northeast quarter of the study area for *T. cristatus*, whereas they were in the southeast part of the study area for *T. marmoratus* (Fig. 3). These areas were highly impacted by the decrease in patch number in *T. cristatus* forecasts, where most patches underwent a decline in interaction flux and thus of their potential of connectivity (Fig. 3). On the other hand, the best-connected areas persisted as such in 2050 and 2100 under the RCP 2.6 scenario for *T. marmoratus* (Fig. 3). The potential of connectivity of habitat patches generally decreased following the reduction in number of habitat patches. For *T. marmoratus*, the pattern of importance of patch connectivity in the southeast compared to the other patches within the study area was no longer so obvious in 2050 under the RCP 8.5 scenario, where some habitat patches reached the connectivity level of other patches in the northeast and west of the study area.

### Two-species combination and assessment of potential changes

**Figure 4:**
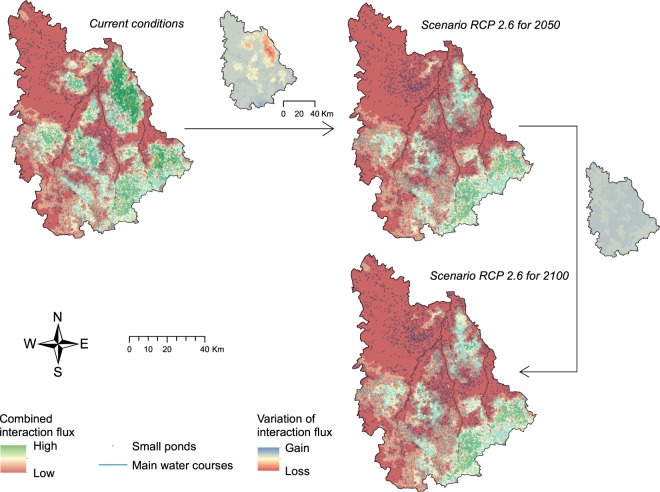
Potential of landscape connectivity resulting from the combination of interaction flux for the two newt species within the study area. Small maps represent the variation of IF between large maps.

Through interpolation and combination of the IFs across the study area, we were able to sum up the potential connectivity for both newt species in a single map, for each condition where we found suitable habitat patches for both species (i.e. current conditions and future scenario RCP 2.6 for 2050 and 2100) (Fig. 4).

We found a high potential of connectivity for newts under current conditions in some patches located in the east, south-eastern and mid-western parts of the study area (Fig. 4). The results under future scenario RCP 2.6 showed an overall contraction of the most connected areas and a reduction in connectivity potential. Most of these changes happened in the north-eastern part of the study area between current conditions and conditions projected for 2050. According to the IFs, connectivity seemed quite stable between 2050 and 2100 in this scenario. Despite the loss of connectivity, we did not actually find any spatial shift of the areas considered best connected according to the IFs within the study area.

## Discussion

The study allowed both large-scale climate tolerances and fine-scale information on land cover to be accounted for, in identifying areas potentially suitable for *T. cristatus* and *T. marmoratus*. We found suitable patches across the whole study area for both species, except in the northwest quarter, where the combination of land-cover-only and climate-only ENMs revealed fewer proper conditions for the presence of newts. Closeness to small ponds took high importance in land cover-only ENMs for both species, and thus in the identification of habitat patches. This makes sense because small ponds are an important habitat for species survival and represent preferential spots for breeding. This emphasizes the relevance of integrating high-resolution data for modelling suitable habitats for species at this scale^65^. Especially for *T. cristatus*, for which climate change might have a high impact, our results emphasized that small ponds are key landscape elements in maintaining newt presence within the study area^66^.

Projections with climate change scenarios revealed a potential impact of warming on habitat patches suitable for newts. Indeed, under RCP 2.6, the scenario leading to the lowest greenhouse gas concentration levels through a “peak-and-decline” succession for 2050 and 2100, we still found a decline in habitat suitability in the study area for *T. cristatus* (from 24% to less than 5%), while habitat suitability for *T. marmoratus* was only slightly impacted. However, with the scenario RCP 8.5, leading to high greenhouse gas concentration levels through rising radiative forcing, habitat patches of *T. cristatus* disappeared from 2050 while those for *T. marmoratus* remained until 2050, and disappeared in 2100. The reduction in suitability for *T. cristatus* is probably related to the fact that it is at the southern limit of its range in our study area. Indeed, it has already been shown that climate change could lead to the displacement of species ranges at higher latitudes, particularly for *T. cristatus*^13,67^.

The calculation of the IF index provides an indication of the local contribution of each patch to overall connectivity within the study area, accounting for both the capacity (i.e. mean of HSI) of the patch and its contribution to overall interactions in the network. Despite a rather high number of suitable patches for both species under current conditions, graph analyses showed that most patches were poorly connected. However, IF calculations and interpolations showed high contribution to overall connectivity for areas in the northeast for *T. cristatus* and in the southeast for *T. marmoratus*, and for a few patches in the southwestern quarter of the study area that showed higher IF for both species. These areas seemed to hold relatively high intrinsic capacity, and to be well connected to other patches. Consequently, these areas could act as source populations within the study area^68^, and to enhance movement in newts, such as seasonal migrations or dispersal. These results could be further developed through genetic analyses to confirm the potential for connectivity among patches^69^.

With climate change scenarios, we observed a decrease in connectivity following the decrease of patch capacity. The variation in IF has made possible to highlight important areas of connectivity for newts and to sum up those potentially threatened by climate warming. In addition, we found that climate change might increase landscape resistance toward newt species. Climate warming is indeed assumed to have direct effects on amphibian ability to move through the landscape, for instance by increasing desiccation risk^5^. However, habitat loss and fragmentation remains a main threat to biodiversity and the related land cover is also subject to constant changes^70^, with impacts on landscape connectivity for amphibians^28,71^. In our forecasts, the increase of landscape resistance is only the result of climate change scenarios, because we did not account for any change in land cover. At this time, there is no coherent scenario of land cover change at the scale of our study area allowing the use of high-resolution data such as the ponds inventory. It would have been opportune to simulate changes in land cover and the evolution of preferential breeding areas for newts, such as ponds, whose maintenance in the landscape depends on both land use and water supply. Indeed, global warming is likely to affect the persistence of wetlands and ponds, and therefore their availability for amphibian reproduction^72^. Still, land use or land cover can possibly buffer impacts of climate warming on landscape connectivity, by ensuring functional connectivity between suitable habitat patches^73^. In fact, Quesnelle *et al*.^74^ found the amount of forest to be more important for amphibians than the amount of wetland, because it provides wintering and summer shelters, and because survival and dispersal is favoured in forested landscapes compared to open areas^75–77^.

The graphs and maps produced in this study are functional tools for environmental planning. The areas identified as suitable and well connected are valuable to the local actors of biodiversity conservation by informing a species conservation strategy; or by highlighting areas where to focus conservation actions, such as prioritizing sites for protection, land control and management, or restoring connectivity.

With the combination of connectivity assessments for both species, we have identified connectivity patches for newts, which it would be interesting to connect in the perspective of climate change. The area identified in the northwestern quarter of the study area was predicted to decrease in connectivity under RCP 2.6. Enhancing the sustaining functionality of the pond network and the connectivity with the surrounding patches might help to buffer this variation, especially by conserving and restoring small ponds and features such as hedgerows or deciduous patches^61,78^ favouring the species’ overwintering, protection against dehydration and seasonal breeding movements. In parallel, relevant actors such as NGOs or other associations in the field of biodiversity protection could systematically bring up the results when consulted on land planning and urban projects, and aware of any risk of habitat destruction or connectivity disruption, particularly in areas identified as sources for these species. Indeed, Matos *et al*.^79^ have found impermeable roads to act as barriers to movements between ponds during both migration and dispersal for *T. cristatus*, whereas smaller and permeable roads increased the size of core areas. Our results can argue for the proposal of measures such as small wildlife crossings to preserve the functionality of the pond network. The inclusion of our results in urban planning documents, for instance, schemes for territorial coherence (as defined by the French urban code) at the level of the administrative department, or local urban development plan (as defined by the French urban code) at the level of municipalities, which have the power to control the destruction of ponds in their respective territories, would improve newt protection. Moreover, maintaining functional connectivity for these low-mobile species would also favour connectivity for more mobile amphibians and other species that depend on small ponds.

Uncertainty in ENM can come from various sources, such as choice of variables, of algorithms, correlation among predictor variables, spatial autocorrelation at species occurrences and transferability of the models in space and time^45^. Here, both land-cover-only and climate-only ENMs obtained satisfying evaluations according to TSS and AUC, which tends to confirm the choice of predictor variables. However, it is noteworthy that the variable “hedges” was not retained during the variable selection procedure whereas it is considered as important for newts^61^. This could be explained by a lack of precision in the data available for the study area, or because this variable is not significant in our study area. We used poorly correlated variables in either climate-only and land-cover-only ENMs and reduced spatial autocorrelation and potential uneven sampling using a single occurrence in each grid-cell containing presence information. We did not find significant spatial autocorrelation on the residuals of our final habitat suitability outputs. In addition, since our models aim to help species conservation in the study area, we estimated areas where individual predictions of the different algorithms could vary, in order to inform practitioners about potential uncertainties. We also provided MESS maps to inform about uncertainty within our projections. We found little extrapolation for current conditions, which allows confidence in transferability of models. However, we found much more extrapolation in future projections for *T. cristatus* than for *T. marmoratus*. Indeed, *T. cristatus* is at the southern limit of its range in our study area and its climate-only ENM was calibrated on data from colder regions than that of *T. marmoratus*. Thus, extrapolation detected for *T. cristatus* under climate warming was the result of projection under non-existent conditions in its current range.

Another point of uncertainty comes from the dispersal distance used to assess the potential connectivity of the species. The choice of this distance for amphibians is not obvious because of little literature on this topic. Dispersal (i.e. an active or passive attempt to move from a natal ⁄ breeding site to another breeding site^80^), generally occurs during the juvenile stages in pond-breeding amphibians^60^, which is why it is difficult to quantify travelled distances. Here we used 1 km as a maximal dispersal distance for newts based on expert opinion, but it could be appropriate to test the effect of a range of dispersal distances^23,81^. The fact that we were not able to include land cover change scenarios at the scale of our study area leads to the unrealistic assumption of no land cover change between current and future conditions^82^. While land cover change is a threat to suitable habitats in the landscape through habitat loss and fragmentation, as explained above, the management of these habitats can also be a lever for species conservation. Moreover, the combination of climate only-ENM and land cover-only ENM outputs assumes that climate and land cover independently influence habitat suitability, which is a source of uncertainty in our framework, as we do not know properly the actual interactions between these two factors. Indeed, climate and land cover might interact through multiple direct and indirect ways, for instance by affecting species response to land cover types under different climatic conditions^83,84^. For all those reasons, our result should be taken with caution and better considered as scenarios than as definite predictions of current and future habitat suitability for the species.

## Conclusion

Combining land cover-based and climate-based ENMs in a single output for graph analyses allowed us to determine patches with high capacity (i.e. mean of HSI) for each species. However, according to Unglaub *et al*.^85^, there is no obvious positive relationship between habitat suitability and performance of populations in *T. cristatus*. Thus, it would be interesting to couple our analyses with local scale population dynamic models or mechanistic models in order to refine our understanding of the relevance of the patches for newt populations. We also determined patches with high interaction flux, which were not necessarily the ones with highest capacity, but which stood apart through a relatively high capacity and high connectivity potential. We consider these as key patches to maintain connectivity and enable the movement of individuals during seasonal migrations or dispersal events through the study area. Thus, after field verifications, these patches could be the subject of protection measures or land control in order to maintain their high potential^86^.

Furthermore, improving patch connectivity of high capacity patches that seemed poorly connected should be considered to promote stepping-stone connectivity allowing a potential range shift, for instance for *T. cristatus*^13,87^. We believe that our framework is a useful and easily reproducible way to combine global climate tolerance of a species with detailed data on species habitats and occurrence when available. It provides information for prioritizing sites for conservation and advises on landscape policies. Our graphs can be reused to simulate potential risks associated with new urban developments, or to select areas in which to create new ponds to promote newt persistence and connectivity.

## Supplementary information

Supplementary information

## Data Availability

The datasets generated during and/or analysed during the current study are available from the corresponding author and under agreement with the naturalist associations that were partners of the study, on reasonable request.
