# Supplementary material for: Habitat patches for newts in the face of climate change: local scale assessment combining niche modelling and graph theory
Source: Sci Rep. 2020 Feb 27;10:3570. doi: 10.1038/s41598-020-60479-4 (PMC7046615; doi:10.1038/s41598-020-60479-4)

Habitat patches for newts in the face of climate change: local scale assessment combining niche modelling and graph theory

Clémentine Préau[1,2,3,*], Frédéric Grandjean, Yann Sellier, Miguel Gailledrat, Romain Bertrand and Francis-Isselin-Nondedeu

[1]Réserve Naturelle Nationale du Pinail, GEREPI, Moulin de Chitré, 86210 Vouneuil-sur-Vienne, France.

[2]Laboratoire Ecologie et Biologie des Interactions – UMR CNRS 7267 Equipe Ecologie Evolution Symbiose, Bâtiment B8-B35, 6, rue Michel Brunet, TSA 51106, 86073 Poitiers Cedex, France.

[3]Département Aménagement et Environnement Ecole Polytechnique de l'Université de Tours, CNRS; UMR CNRS 7324 CITERES; 33-35 Allée Ferdinand de Lesseps, 37200 Tours, France.

[*]corresponding author, clementine.preau@univ-poitiers.fr

ESM 1: Workflow of analysis

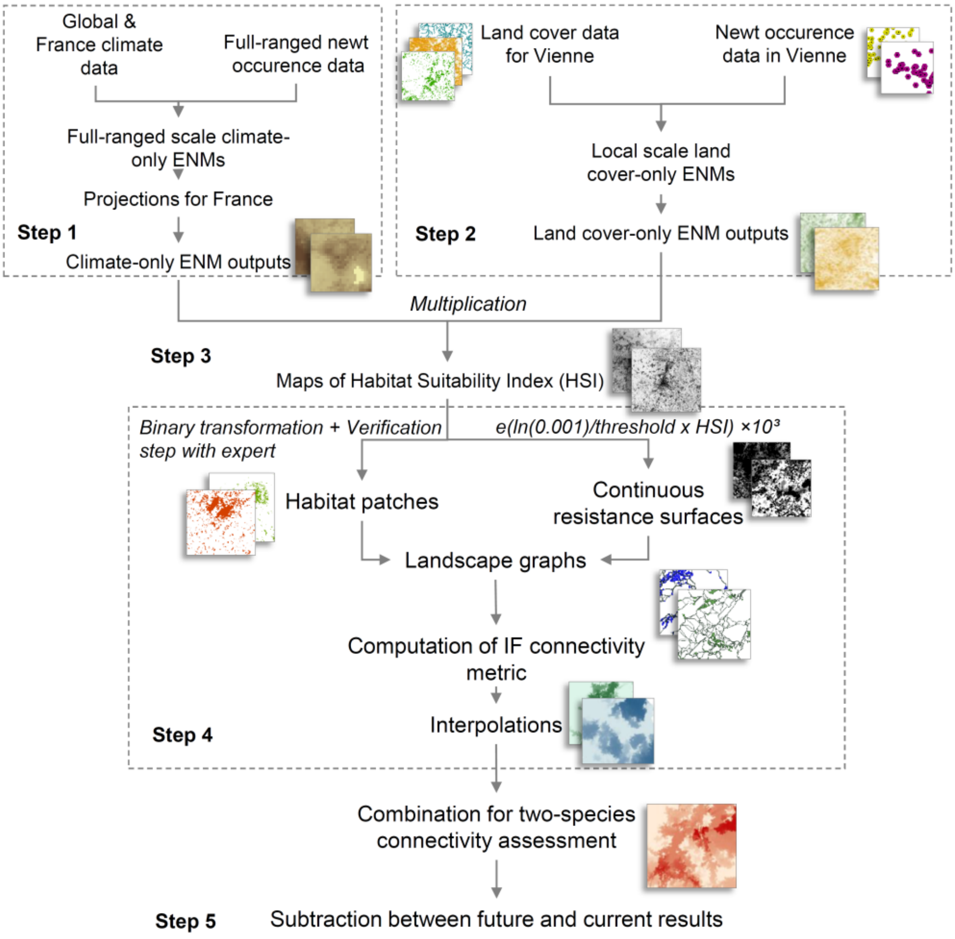

ESM 2: Description of variables, correlation and selection of variables

Variable description:

Model	Original resolution	Variable	Description	Source	*Triturus cristatus*	*Triturus marmoratus*
Climate-only ENMs	10x10 km	BIO01	Annual Mean Temperature	Worldclim V.2		X
		BIO02	Mean Diurnal Range (Mean of monthly (max temp - min temp))			X
		BIO03	Isothermality (BIO2/BIO7) (* 100)			
		BIO04	Temperature Seasonality (standard deviation *100)			X
		BIO05	Max Temperature of Warmest Month		X	
		BIO06	Min Temperature of Coldest Month			
		BIO07	Temperature Annual Range (BIO5-BIO6)		X	
		BIO08	Mean Temperature of Wettest Quarter		X	X
		BIO09	Mean Temperature of Driest Quarter			X
		BIO10	Mean Temperature of Warmest Quarter			
		BIO11	Mean Temperature of Coldest Quarter		X	
		BIO12	Annual Precipitation		X	
		BIO13	Precipitation of Wettest Month			
		BIO14	Precipitation of Driest Month			
		BIO15	Precipitation Seasonality (Coefficient of Variation)		X	X
		BIO16	Precipitation of Wettest Quarter			
		BIO17	Precipitation of Driest Quarter		X	
		BIO18	Precipitation of Warmest Quarter			X
		BIO19	Precipitation of Coldest Quarter			X
Climate-only ENMs	1x1 km	BIO01	Annual Mean Temperature	Bertrand et al. (2016), Haylock et al. (2008), Oldenborgh et al. (2013)		X
		BIO02	Mean Diurnal Range (Mean of monthly (max temp - min temp))			X
		BIO04	Temperature Seasonality (standard deviation *100)			X
		BIO05	Max Temperature of Warmest Month		X	
		BIO07	Temperature Annual Range (BIO5-BIO6)		X	

		BIO08	Mean Temperature of Wettest Quarter		X	X
		BIO09	Mean Temperature of Driest Quarter			X
		BIO11	Mean Temperature of Coldest Quarter		X	
		BIO12	Annual Precipitation		X	
		BIO15	Precipitation Seasonality (Coefficient of Variation)		X	X
		BIO17	Precipitation of Driest Quarter		X	
		BIO18	Precipitation of Warmest Quarter			X
		BIO19	Precipitation of Coldest Quarter			X
Land cover-only ENMs	25x25m	Elevation	Elevation in m	IGN BD ALTI	X	X
	10x10m	Coniferous forests	Surface-Compacity Index	CESBIO		
	10x10m	Broad-leaved forests	Surface-Compacity Index	CESBIO	X	X
		Headges	Surface-Compacity Index	IGN BD TOPO		
	10x10m	Woody moorlands	Surface-Compacity Index	CESBIO		
	10x10m	Beaches, dunes and sand	Surface-Compacity Index	CESBIO		
	10x10m	Natural grasslands	Surface-Compacity Index	CESBIO		
	10x10m	Intensive grasslands	Surface-Compacity Index	CESBIO		
	10x10m	Vineyards	Surface-Compacity Index	CESBIO		
	10x10m	Crops	Surface-Compacity Index	CESBIO		X
	50x50m	Water courses	Distance to the closest in m	IGN BD TOPO	X	
	50x50m	Large ponds	Surface-Compacity Index	Vienne Nature inventory from SCAN 25, IGN http://professionnels.ign.fr/scan25, and orthophotos, IGN http://professionnels.ign.fr/bdortho		
	50x50m	Small ponds	Distance to the closest in m	Vienne Nature inventory from SCAN 25, IGN http://professionnels.ign.fr/scan25, and orthophotos, IGN http://professionnels.ign.fr/bdortho	X	X
	50x50m	Springs	Distance to the closest in m	Vienne Nature inventory from	X	X

				SCAN 25, IGN http://professionnels.ign.fr/scan25, and orthophotos, IGN http://professionnels.ign.fr/bdortho		
	20x20m	Urban areas	% of imperviousness	Copernicus High resolution		

Correlation among bioclimatic variables across *T. cristatus* range (red boxes are groups of uncorrelated variables considering a distance of 0.3 as threshold):

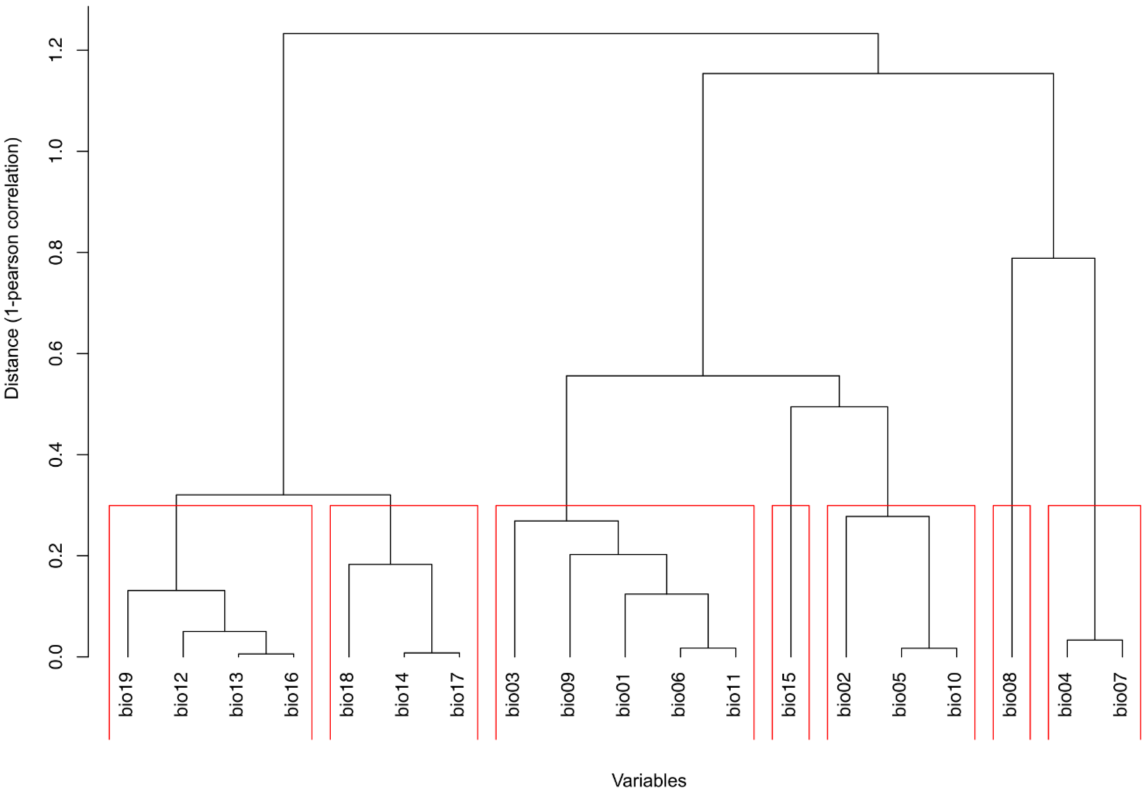

Correlation among bioclimatic variables across *T. marmoratus* range (red boxes are groups of uncorrelated variables considering a distance of 0.3 as threshold):

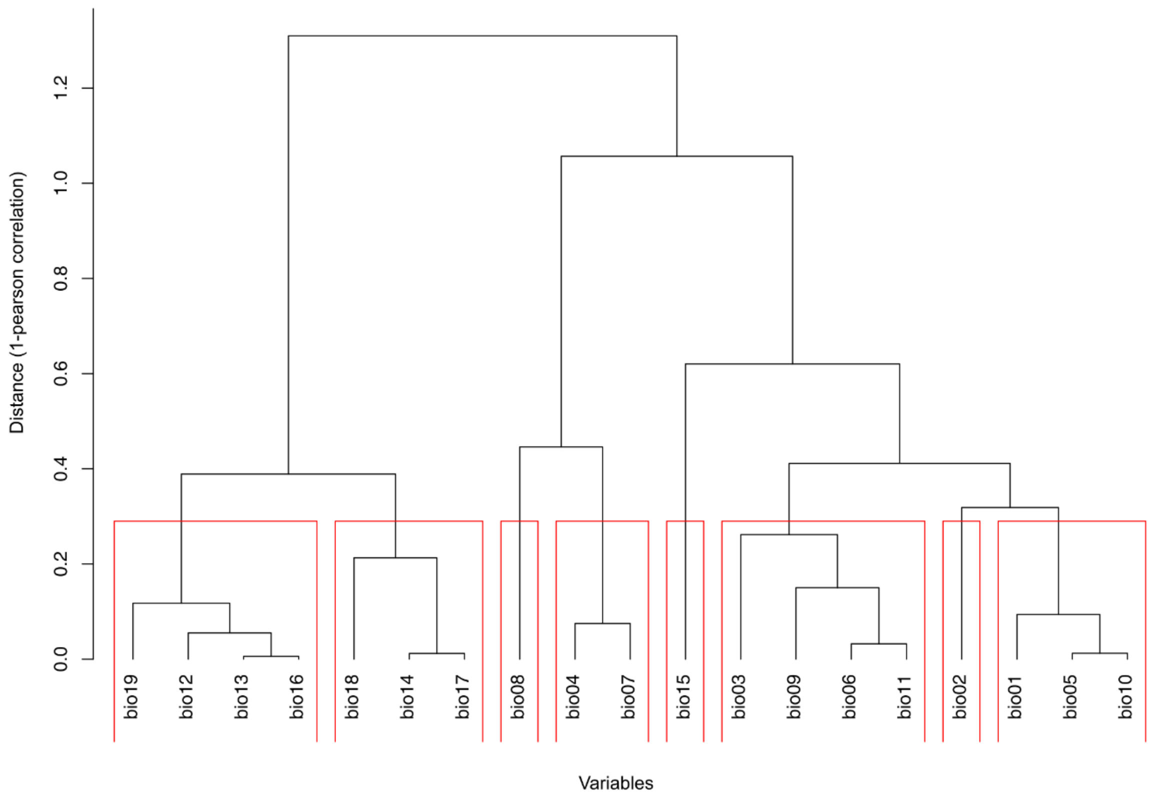

Correlation among land-cover variables across the administrative department of Vienne:

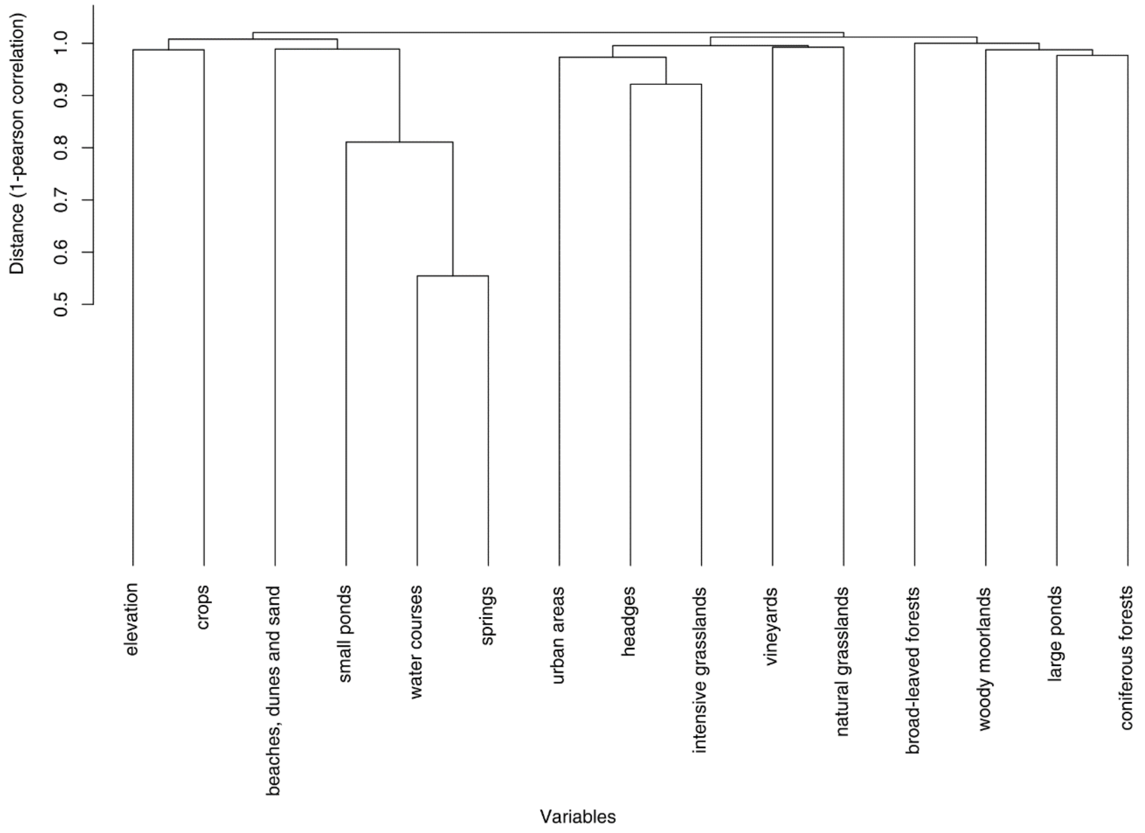

Bertrand R, Riofrio-Dillon G, Lenoir J et al (2016) Ecological constraints increase the climatic debt in forests. Nature communications 7:12643

Haylock MR, Hofstra N, Klein Tank AMG, Klok EJ, Jones PD, New M (2008) A European daily high-resolution gridded data set of surface temperature and precipitation for 1950–2006. Journal of Geophysical Research: Atmospheres 113:D20

Oldenborgh GJv, Reyes FJD, Drijfhout SS, Hawkins E (2013) Reliability of regional climate model trends. Environmental Research Letters 8(1):014055

* Current and future climate conditions were extracted from 1-km2 grids covering France. These climate grids were computed by downscaling two climate datasets having complementary spatiotemporal characteristics (using the delta method; e.g., Tabor and Williams (2010)). First, we computed the climate anomalies between coarse European climate grids for the 2000–2016 period (Haylock et al. 2008) or for the 2051–2060 period (Oldenborgh et al. 2013) and the 1961–1990 period (i.e., the climate reference period) as absolute and relative differences for temperature and precipitation, respectively. Second, we resampled these current and future anomaly grids through bilinear interpolation to achieve 1-km2 grids covering the French territory. Third, we added the 1-km2 climate anomaly grids to a second set of 1-km2 French climate grids of temperature and precipitation averaged over the 1961–1990 period in order to achieve an accurate estimation of the climate conditions for the 2000–2016 period. The second set of 1-km2 French climate grids used for this step are part of a high spatio-temporal resolution climate dataset computed through a modeling approach predicting temperatures (R2 = 0.93 and root-meansquare deviation [RMSD] = 0.56 for 13,620 independent temperature observations) and precipitation (R2 = 0.83 and RMSD = 132 for 17,865 independent precipitation observations) from the statistical link existing between meteorological observations (from the national agency for meteorology and climate MeteoFrance) and a set of geographical and physiographical variables (e.g., Bertrand et al. (2016)).

ESM 3: Model parameters

Parameters for climate-only ENM for *T. cristatus*:

myBiomod_options<-BIOMOD_ModelingOptions(GLM = list(type = 'quadratic', interaction.level = 3, myFormula = NULL, test = 'AIC', family = binomial(link = 'logit'), mustart = 0.5, control = glm.control(epsilon = 1e-08, maxit = 50, trace = FALSE)),

GBM = list(distribution = 'bernoulli', n.trees = 4245, interaction.depth = 4, n.minobsinnode = 13, shrinkage = 0.09554579, bag.fraction = 0.5, train.fraction = 1, cv.folds = 3, keep.data = FALSE, verbose = FALSE, perf.method = 'cv'),

GAM = list(algo = 'GAM_mgcv', type = 's_smoother', k = -1, interaction.level = 3, myFormula = NULL, family = binomial(link = 'logit'), method = 'ML', optimizer = c('outer','newton'), select = FALSE, knots = NULL, paraPen = NULL, control = list(nthreads = 1, irls.reg = 0, epsilon = 1e-07, maxit = 200, trace = FALSE, mgcv.tol = 1e-07, mgcv.half = 15, rank.tol = 1.49011611938477e-08, nlm = list(ndigit=7, gradtol=1e-06, stepmax=2, steptol=1e-04, iterlim=200, check.analyticals=0), optim = list(factr=1e+07), newton = list(conv.tol=1e-06, maxNstep=5, maxSstep=2, maxHalf=30, use.svd=0), outerPIsteps = 0, idLinksBases = TRUE, scalePenalty = TRUE, keepData = FALSE, edge.correct = FALSE)),

CTA = list(method = 'class', parms = 'default', cost = NULL, control = list(xval = 5, minbucket = 5, minsplit = 5, cp = 0.0002722676, maxdepth = 25)),

ANN = list(NbCV = 5, size = 9, decay = 0.1996269, rang = 0.1, maxit = 200),

FDA = list(method = 'mars', nprune = 11),

MARS = list(type = 'simple', interaction.level = 3, myFormula = NULL, nk = NULL, penalty = 2, thresh = 0.001, nprune = 14, pmethod = 'backward'),

RF = list(do.classif = TRUE, ntree = 500, mtry = 3, nodesize = 5, maxnodes = NULL),

MAXENT.Phillips = list(memory_allocated = 512, background_data_dir = 'default', maximumbackground = 'default', maximumiterations = 200, visible = FALSE, linear = FALSE, quadratic = TRUE, product = FALSE, threshold = FALSE, hinge = FALSE, lq2lqptthreshold = 80, l2lqthreshold = 10, hingethreshold = 15, beta_threshold = -1, beta_categorical = -1, beta_lqp = -1, beta_hinge = -1, betamultiplier = 1, defaultprevalence = 0.5))

Parameters for climate -only ENM for *T. marmoratus*:

myBiomod_options<-BIOMOD_ModelingOptions(GLM = list(type = 'quadratic', interaction.level = 3, myFormula = NULL, test = 'AIC', family = binomial(link = 'logit'), mustart = 0.5, control = glm.control(epsilon = 1e-08, maxit = 50, trace = FALSE)),

GBM = list(distribution = 'bernoulli', n.trees = 2853 , interaction.depth = 7, n.minobsinnode = 25, shrinkage = 0.09729011 , bag.fraction = 0.5, train.fraction = 1, cv.folds = 3, keep.data = FALSE, verbose = FALSE, perf.method = 'cv'),

GAM = list(algo = 'GAM_mgcv', type = 's_smoother', k = -1, interaction.level = 3, myFormula = NULL, family = binomial(link = 'logit'), method = 'GCV.Cp', optimizer = c('outer','newton'), select = FALSE, knots = NULL, paraPen = NULL, control = list(nthreads = 1, irls.reg = 0, epsilon = 1e-07, maxit = 200, trace = FALSE, mgcv.tol = 1e-07, mgcv.half = 15, rank.tol = 1.49011611938477e-08, nlm = list(ndigit=7, gradtol=1e-06, stepmax=2, steptol=1e-04, iterlim=200, check.analyticals=0), optim = list(factr=1e+07), newton = list(conv.tol=1e-06, maxNstep=5, maxSstep=2, maxHalf=30, use.svd=0), outerPIsteps = 0, idLinksBases = TRUE, scalePenalty = TRUE, keepData = FALSE, edge.correct = FALSE)),

CTA = list(method = 'class', parms = 'default', cost = NULL, control = list(xval = 5, minbucket = 5, minsplit = 5, cp = 0.001217203, maxdepth = 25)),

ANN = list(NbCV = 5, size = 7, decay = 0.1122347, rang = 0.1, maxit = 200),

FDA = list(method = 'mars', nprune = 10),

MARS = list(type = 'simple', interaction.level = 3, myFormula = NULL, nk = NULL, penalty = 2, thresh = 0.001, nprune = 13, pmethod = 'backward'),

RF = list(do.classif = TRUE, ntree = 500, mtry = 1, nodesize = 5, maxnodes = NULL),

MAXENT.Phillips = list(memory_allocated = 512, background_data_dir = 'default', maximumbackground = 'default', maximumiterations = 200, visible = FALSE, linear = FALSE, quadratic = TRUE, product = FALSE, threshold = FALSE, hinge = FALSE, lq2lqptthreshold = 80, l2lqthreshold = 10, hingethreshold = 15, beta_threshold = -1, beta_categorical = -1, beta_lqp = -1, beta_hinge = -1, betamultiplier = 1, defaultprevalence = 0.5))

Parameters for land cover-only ENM for *T. cristatus*:

myBiomod_options<-BIOMOD_ModelingOptions(GLM = list(type = 'quadratic', interaction.level = 0, myFormula = NULL, test = 'AIC', family = binomial(link = 'logit'), mustart = 0.5, control = glm.control(epsilon = 1e-08, maxit = 50, trace = FALSE)),

GBM = list(distribution = 'bernoulli', n.trees = 293, interaction.depth = 7, n.minobsinnode = 7, shrinkage = 0.194193, bag.fraction = 0.5, train.fraction = 1, cv.folds = 3, keep.data = FALSE, verbose = FALSE, perf.method = 'cv'),

GAM = list(algo = 'GAM_mgcv', type = 's_smoother', k = -1, interaction.level = 0, myFormula = NULL, family = binomial(link = 'logit'), method = 'ML', optimizer = c('outer','newton'), select = TRUE, knots = NULL, paraPen = NULL, control = list(nthreads = 1, irls.reg = 0, epsilon = 1e-07, maxit = 200, trace = FALSE, mgcv.tol = 1e-07, mgcv.half = 15, rank.tol = 1.49011611938477e-08, nlm = list(ndigit=7, gradtol=1e-06, stepmax=2, steptol=1e-04, iterlim=200, check.analyticals=0), optim = list(factr=1e+07), newton = list(conv.tol=1e-06, maxNstep=5, maxSstep=2, maxHalf=30, use.svd=0), outerPIsteps = 0, idLinksBases = TRUE, scalePenalty = TRUE, keepData = FALSE, edge.correct = FALSE)),

CTA = list(method = 'class', parms = 'default', cost = NULL, control = list(xval = 5, minbucket = 5, minsplit = 5, cp = 0.03278689, maxdepth = 25)),

ANN = list(NbCV = 5, size = 19, decay = 2.10529, rang = 0.1, maxit = 200),

FDA = list(method = 'mars', nprune = 9),

MARS = list(type = 'simple', interaction.level = 3, myFormula = NULL, nk = NULL, penalty = 2, thresh = 0.001, nprune = 7, pmethod = 'backward'),

RF = list(do.classif = TRUE, ntree = 500, mtry = 1, nodesize = 5, maxnodes = NULL),

MAXENT.Phillips = list(memory_allocated = 512, background_data_dir = 'default', maximumbackground = 'default', maximumiterations = 200, visible = FALSE, linear = TRUE, quadratic = TRUE, product = TRUE, threshold = TRUE, hinge = TRUE, lq2lqptthreshold = 80, l2lqthreshold = 10, hingethreshold = 15, beta_threshold = -1, beta_categorical = -1, beta_lqp = -1, beta_hinge = -1, betamultiplier = 1, defaultprevalence = 0.5))

Parameters for land cover-only ENM for *T. marmoratus*:

myBiomod_options<-BIOMOD_ModelingOptions(GLM = list(type = 'quadratic', interaction.level = 0, myFormula = NULL, test = 'AIC', family = binomial(link = 'logit'), mustart = 0.5, control = glm.control(epsilon = 1e-08, maxit = 50, trace = FALSE)),

GBM = list(distribution = 'bernoulli', n.trees = 1236, interaction.depth = 1, n.minobsinnode = 13, shrinkage = 0.03417332, bag.fraction = 0.5, train.fraction = 1, cv.folds = 3, keep.data = FALSE, verbose = FALSE, perf.method = 'cv'),

GAM = list(algo = 'GAM_mgcv', type = 's_smoother', k = -1, interaction.level = 0, myFormula = NULL, family = binomial(link = 'logit'), method = 'GCV.Cp', optimizer = c('outer','newton'), select = FALSE, knots = NULL, paraPen = NULL, control = list(nthreads = 1, irls.reg = 0, epsilon = 1e-07, maxit = 200, trace = FALSE, mgcv.tol = 1e-07, mgcv.half = 15 , rank.tol = 1.49011611938477e-08, nlm = list(ndigit=7, gradtol=1e-06, stepmax=2, steptol=1e-04, iterlim=200, check.analyticals=0), optim = list(factr=1e+07), newton = list(conv.tol=1e-06, maxNstep=5, maxSstep=2, maxHalf=30, use.svd=0), outerPIsteps = 0, idLinksBases = TRUE, scalePenalty = TRUE, keepData = FALSE, edge.correct = FALSE)),

CTA = list(method = 'class', parms = 'default', cost = NULL, control = list(xval = 5, minbucket = 5, minsplit = 5, cp = 0.005527638, maxdepth = 25)),

ANN = list(NbCV = 5, size = 14, decay = 0.00022404, rang = 0.1, maxit = 200),

FDA = list(method = 'mars', nprune = 2),

MARS = list(type = 'simple', interaction.level = 0, myFormula = NULL, nk = NULL, penalty = 2, thresh = 0.001, nprune = 2, pmethod = 'backward'),

RF = list(do.classif = TRUE, ntree = 500, mtry = 1, nodesize = 5, maxnodes = NULL),

MAXENT.Phillips = list(memory_allocated = 512, background_data_dir = 'default', maximumbackground = 'default', maximumiterations = 200, visible = FALSE, linear = TRUE, quadratic = FALSE, product = TRUE, threshold = TRUE, hinge =TRUE, lq2lqptthreshold = 80, l2lqthreshold = 10, hingethreshold = 15, beta_threshold = -1, beta_categorical = -1, beta_lqp = -1, beta_hinge = -1, betamultiplier = 5, defaultprevalence = 0.5))

ESM 4: Range of values of climate data

Range of values in Worldclim data within the range of the species compared to dataset (calculated for France) used within the study area:

		10x10 km (Worldclim data)			1x1km (Data used within the study area)		
Triturus cristatus model		Maximum	Median	Minimum	Maximum	Median	Minimum
bio05	Max temperature of warmest month	43.72	22.27	0.00	25.00	24.18	22.43
bio07	Temperature annual range	55.01	34.96	0.00	21.88	21.02	19.55
bio08	Mean temperature of wettest quarter	24.03	12.99	-11.50	11.54	10.06	6.85
bio11	Mean temperature of coldest quarter	14.46	-5.05	-23.31	6.98	6.15	5.53
bio12	Annual precipitation	3625.00	559.00	83.00	1184.47	749.08	630.08
bio15	Precipitation seasonality	106.06	32.84	4.42	23.44	15.33	11.94
bio17	Precipitation of driest quarter	510.00	84.00	0.00	233.25	164.55	132.78

		10x10 km (Worldclim data)			1x1km (Data used within the study area)		
Triturus marmoratus model		Maximum	Median	Minimum	Maximum	Median	Minimum
bio01	Annual mean T°	22.97	9.28	-5.413208	13.24	12.61	11.95
bio02	Mean diurnal range of monthly T°	14.73	8.29	2.25	7.95	7.30	5.83
bio04	Temperature seasonality	936.41	671.35	191.55	537.61	528.09	510.69
bio08	Mean temperature of wettest quarter	23.43	12.60	-11.50	11.54	10.06	6.85
bio09	Mean temperature of driest quarter	33.34	5.02	-10.84	19.53	18.83	6.65
bio15	Precipitation seasonality	96.56	28.92	4.42	23.44	15.33	11.94
bio18	Precipitation of warmest quarter	653.00	199.00	4.00	236.72	173.49	137.88
bio19	Precipitation of coldest quarter	1142.00	162.00	22.00	327.26	194.84	156.15

ESM 5: Pearson correlation between bioclimatic variables used for model calibration and bioclimatic variables used for model projection

	p-value	R^2
BIO01_wc$BIO01_France	<2e-16 ***	0.96164775
BIO02_wc$BIO02_France	<2e-16 ***	0.20971152
BIO04_wc$BIO04_France	<2e-16 ***	0.7936604
BIO05_wc$BIO05_France	<2e-16 ***	0.88012448
BIO07_wc$BIO07_France	<2e-16 ***	0.60009021
BIO08_wc$BIO08_France	<2e-16 ***	0.37172751
BIO09_wc$BIO09_France	<2e-16 ***	0.62105259
BIO11_wc$BIO11_France	<2e-16 ***	0.93903872
BIO12_wc$BIO12_France	<2e-16 ***	0.58788455
BIO15_wc$BIO15_France	<2e-16 ***	0.79749847
BIO16_wc$BIO16_France	<2e-16 ***	0.54221397
BIO17_wc$BIO17_France	<2e-16 ***	0.66846322
BIO18_wc$BIO18_France	<2e-16 ***	0.73206693
BIO19_wc$BIO19_France	<2e-16 ***	0.52491329

ESM 6: Coefficient of variation between single models

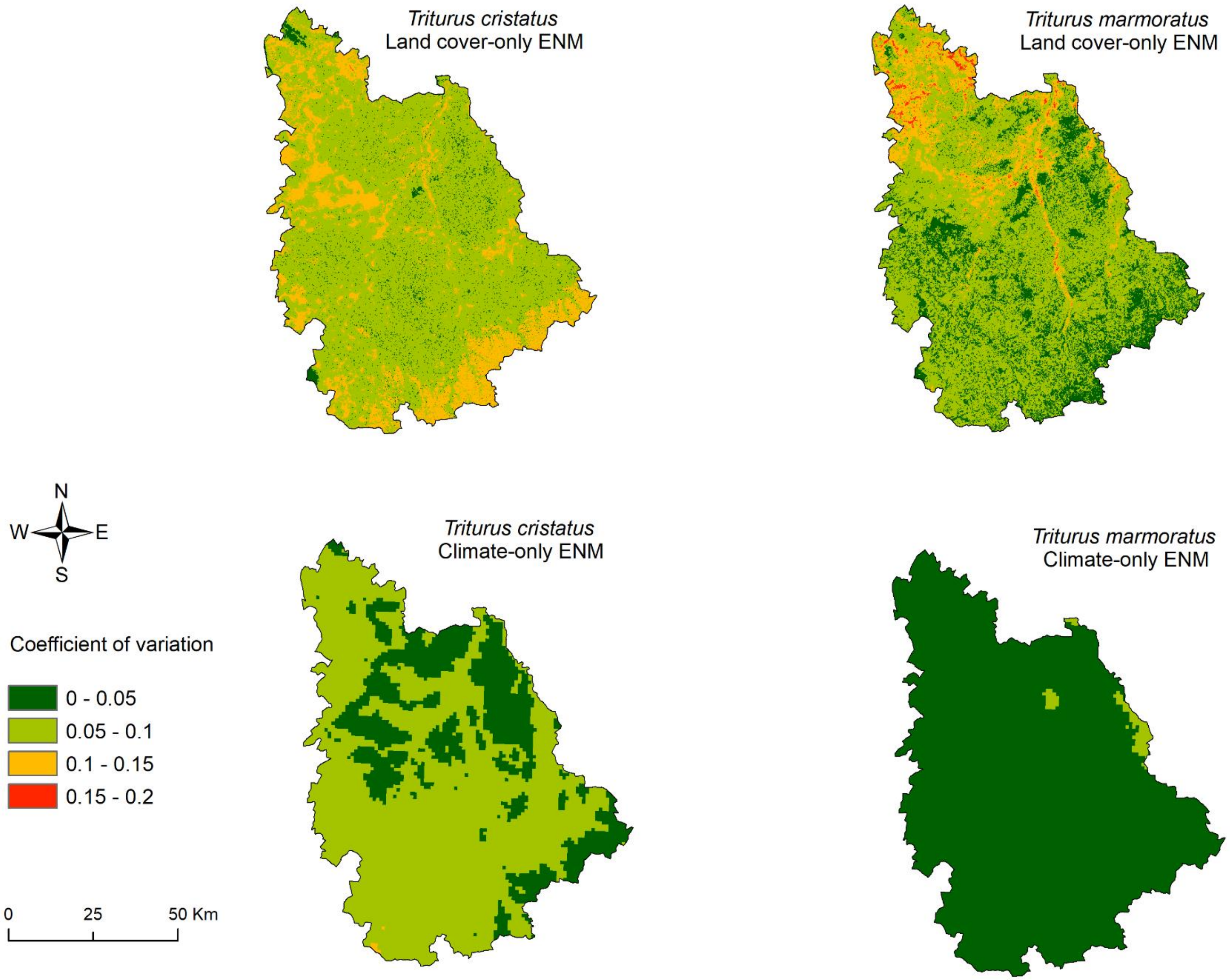

ESM 7: MESS analysis

Results of MESS analysis for current conditions:

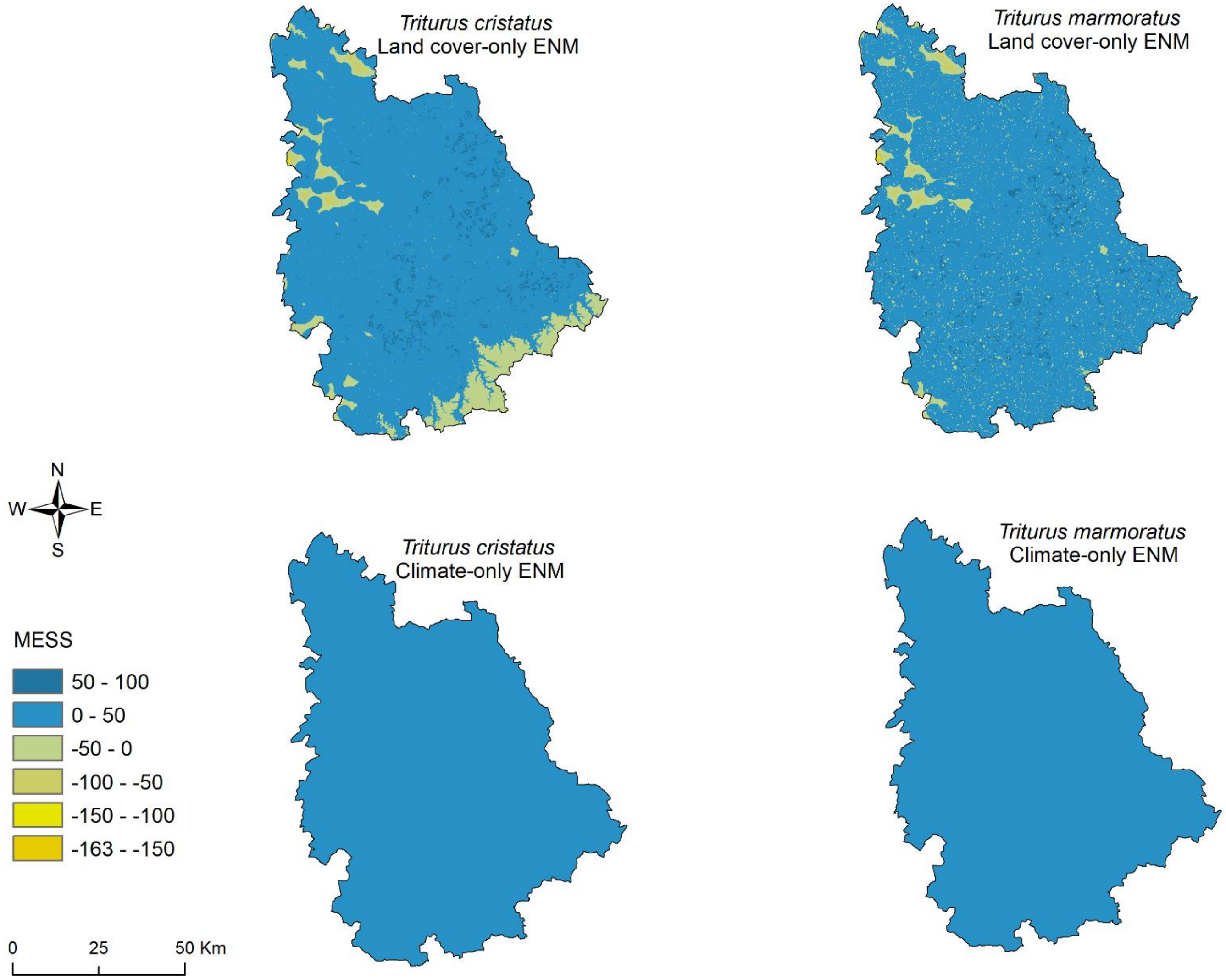

Results of MESS analysis for future conditions, for *T. marmoratus*:

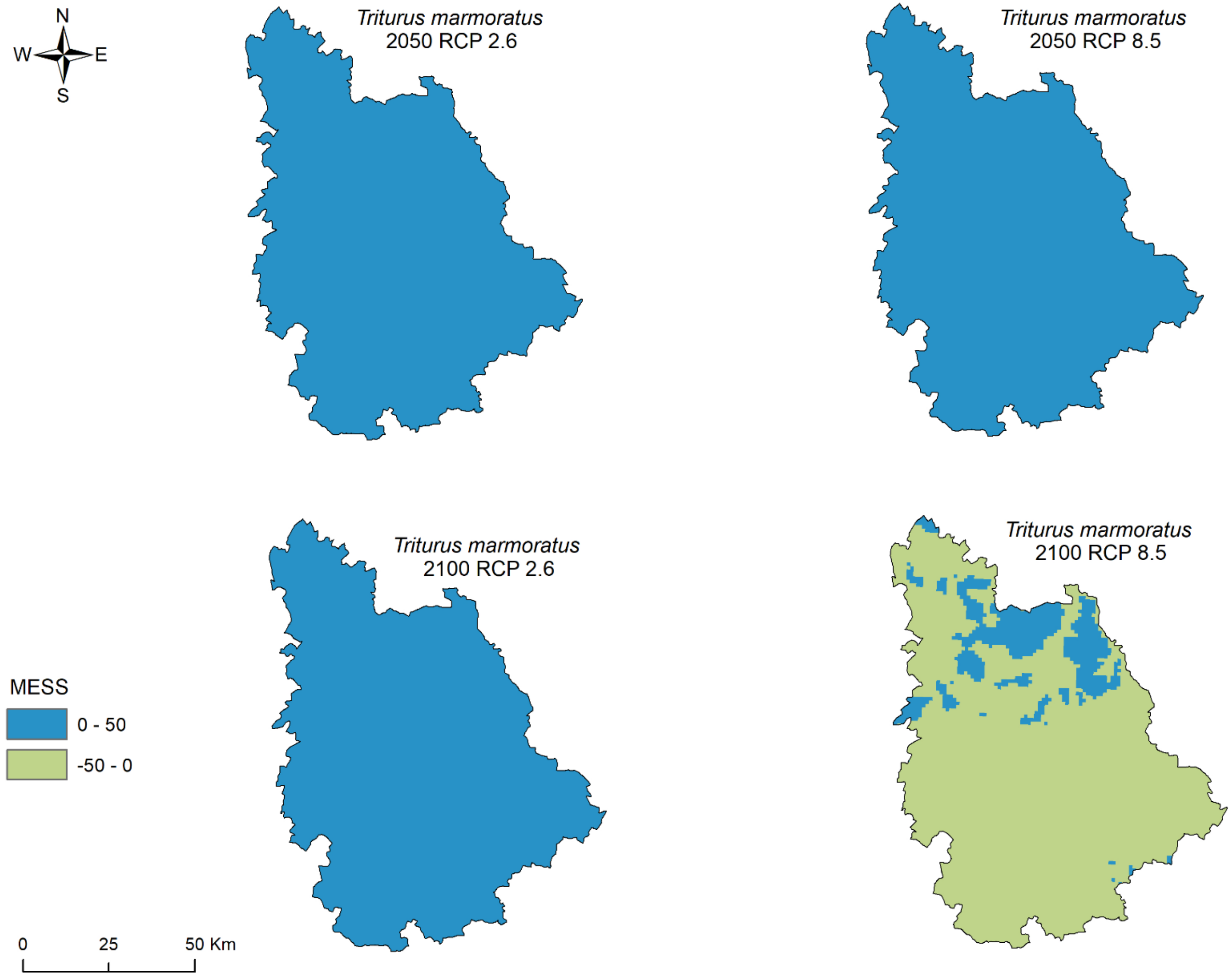

Results of MESS analysis for future conditions, for *T. cristatus*:

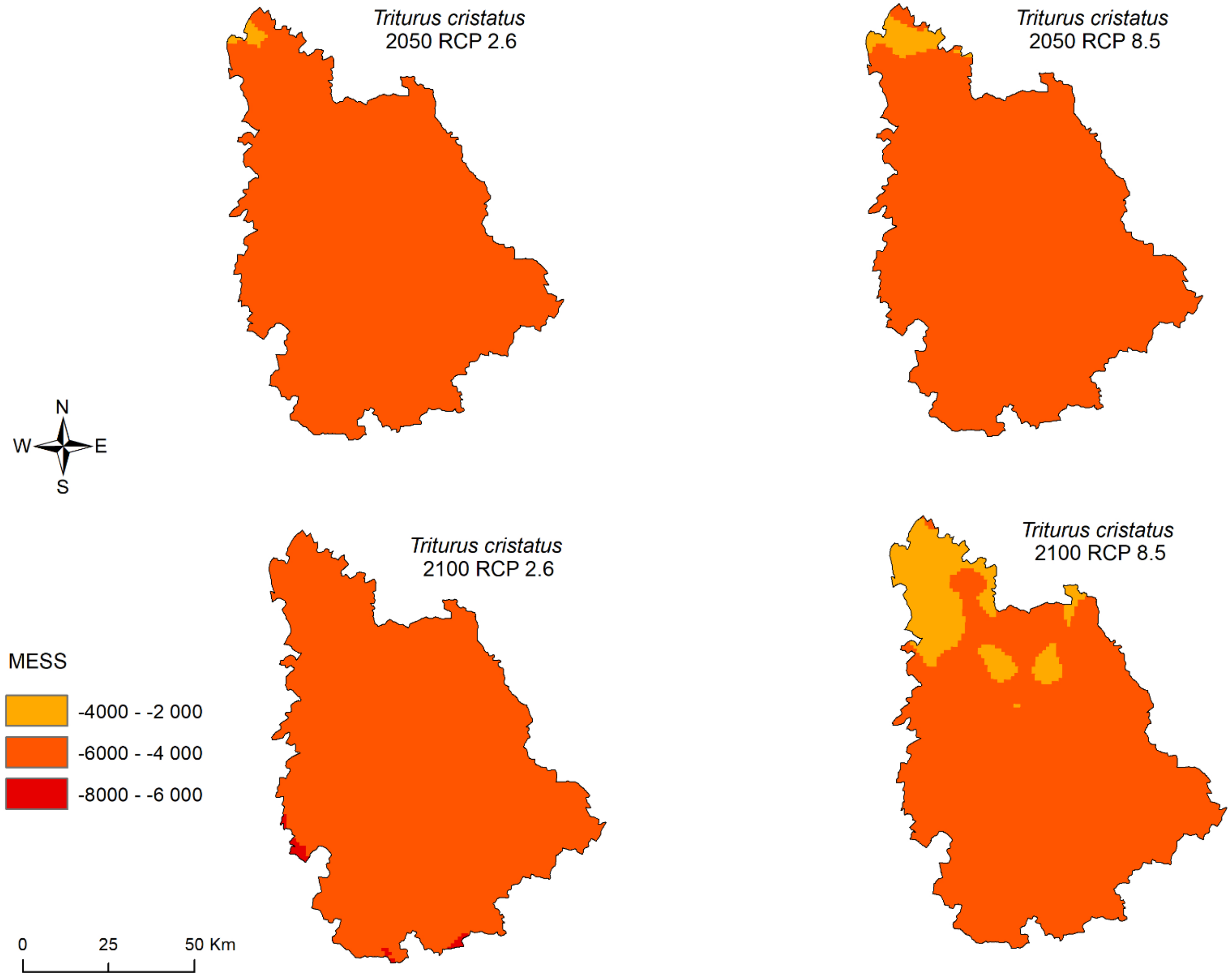

ESM 8: Variable importance

Relative importance of variables in climate-only ENMs:

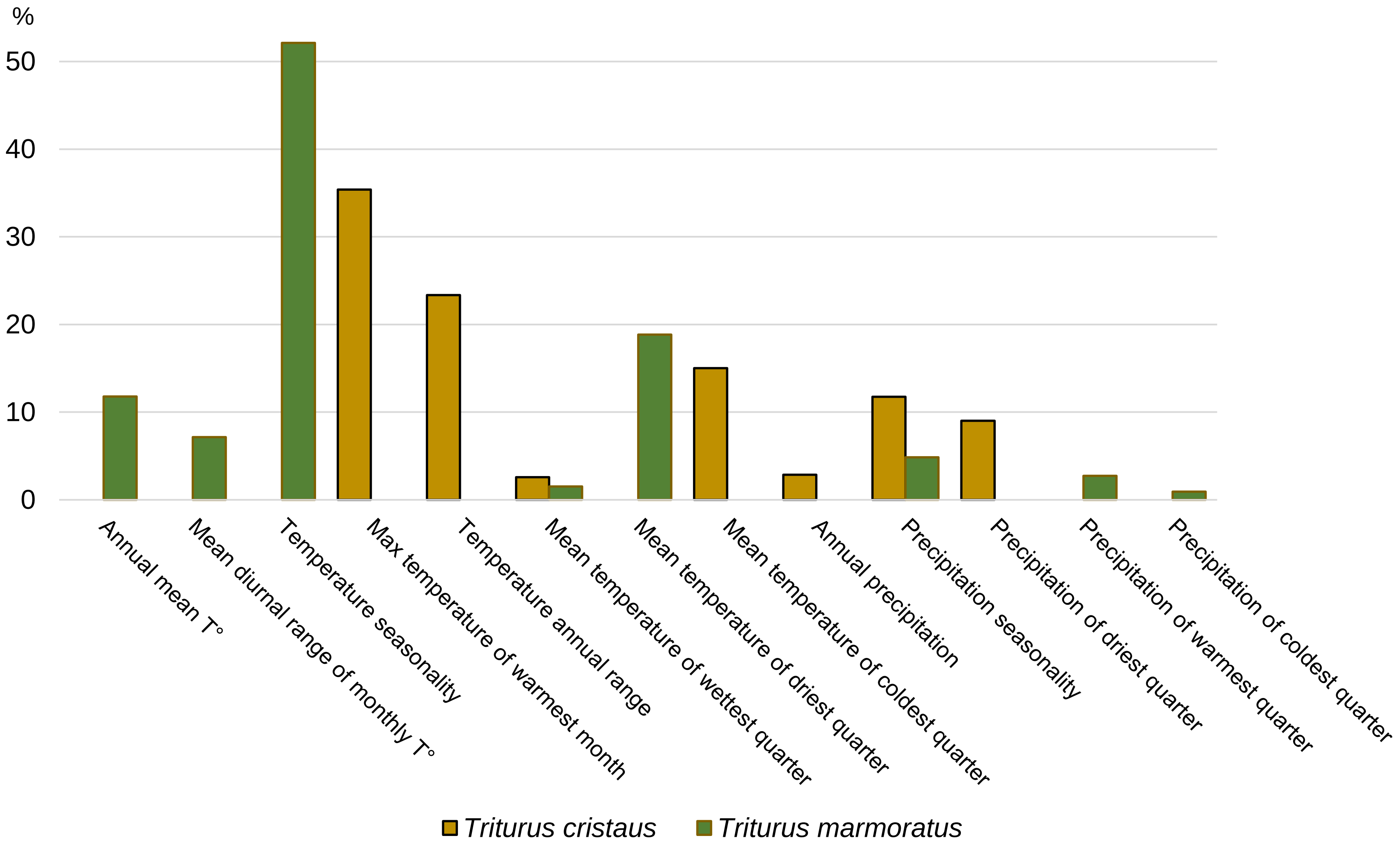

Relative importance of variables in land cover-only ENMs:

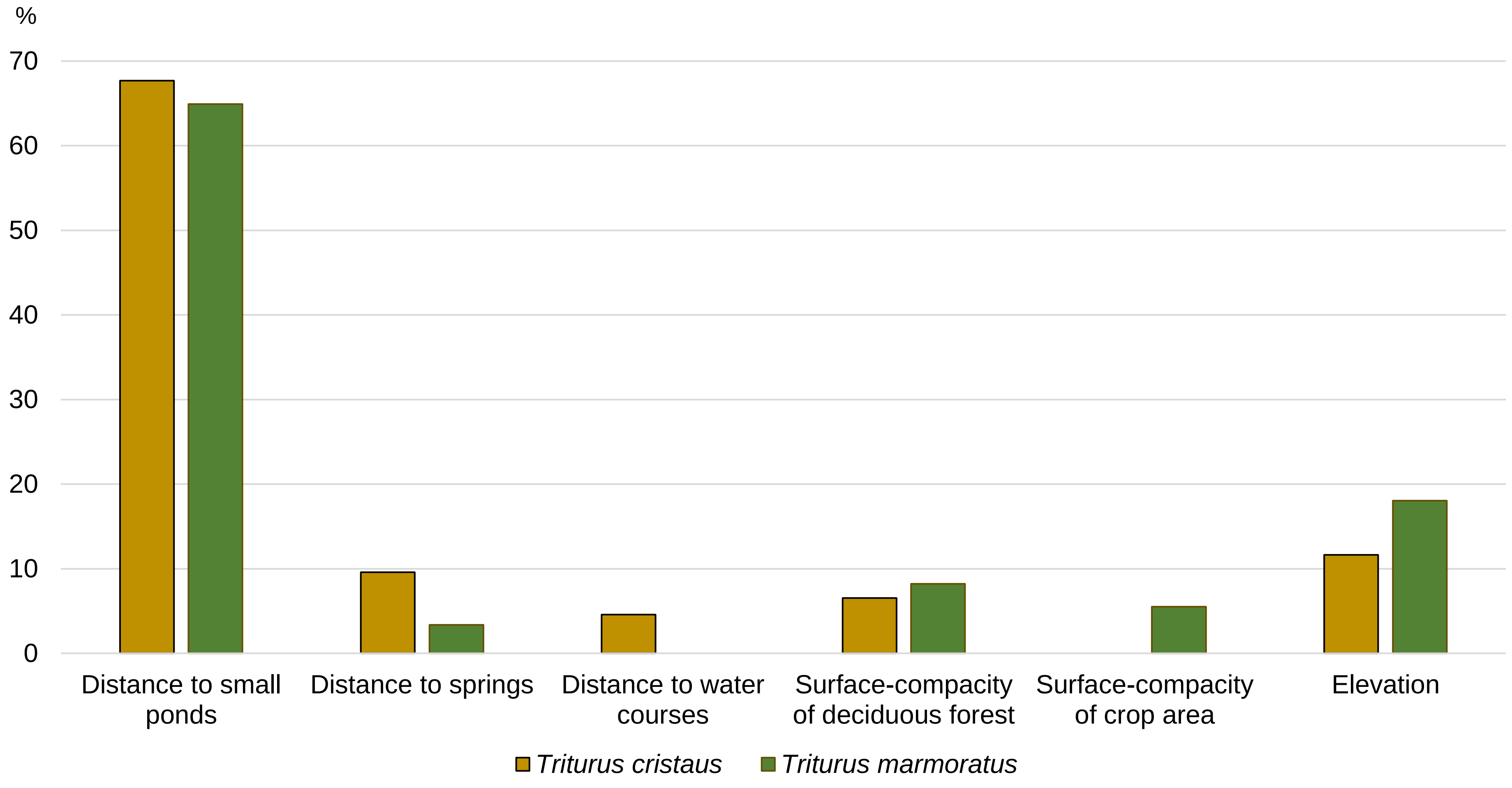

ESM 9: Histograms showing the evolution of suitable habitat for current conditions and future scenarios in places where the species are currently present

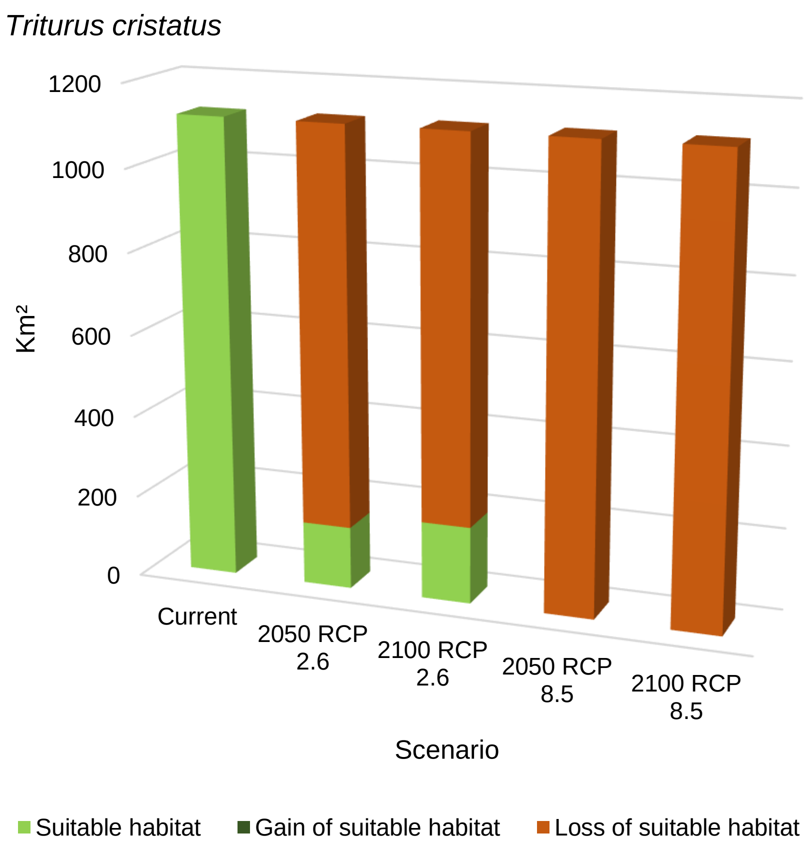

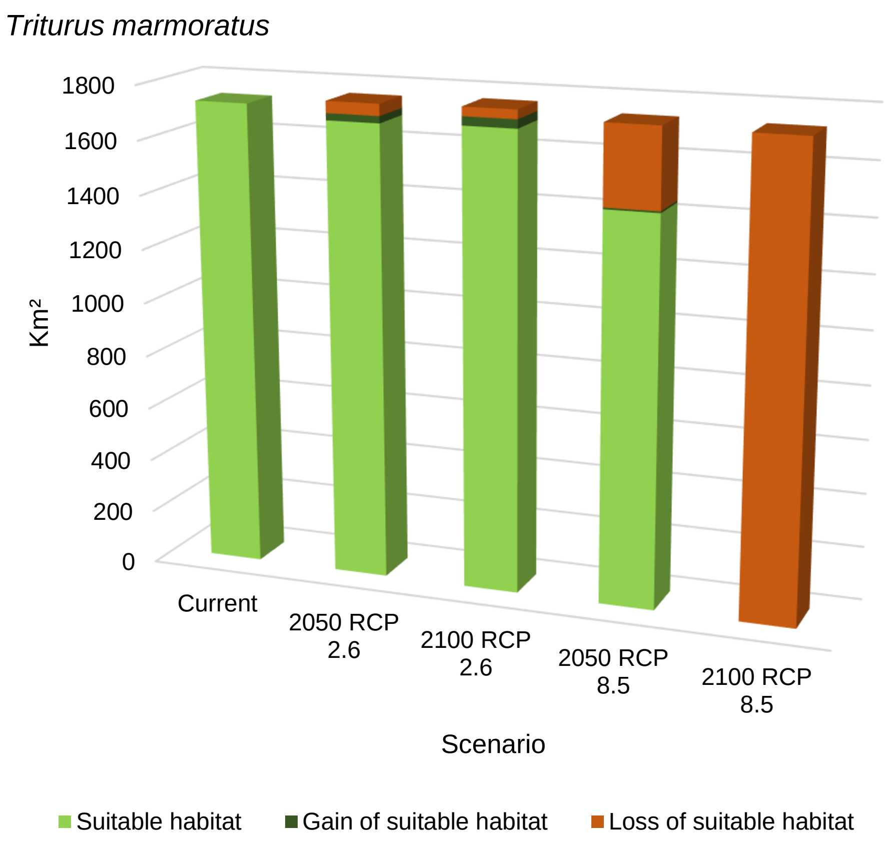

ESM 10: Global Moran's I

Histogram of Moran's I random permutations against global Moran's I value (red bar), on *T. cristatus'* final habitat suitability residuals (p-value = 0.118):

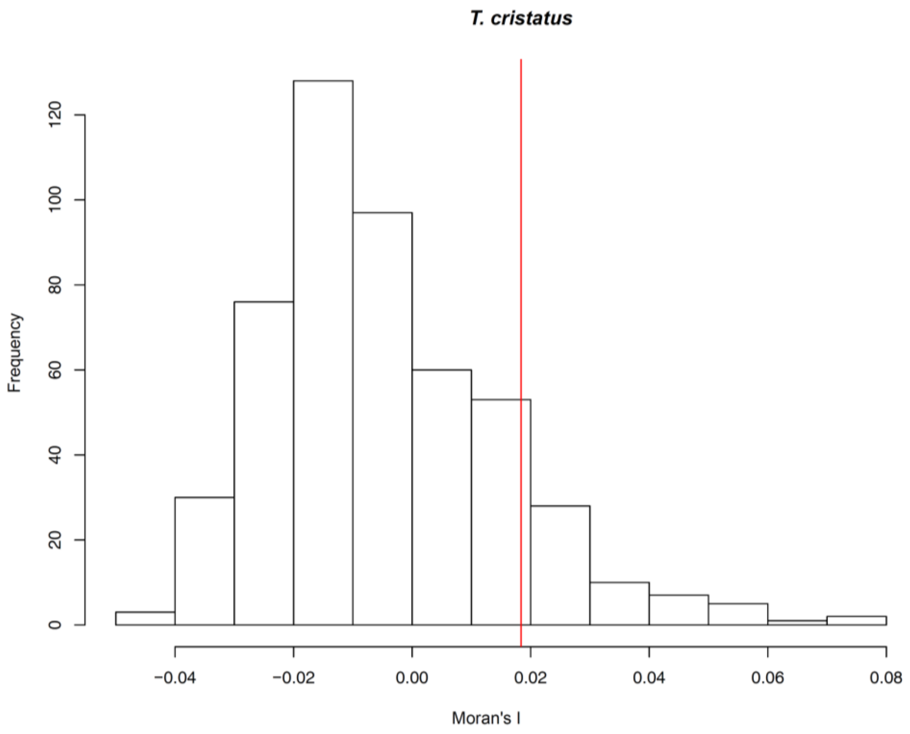

Histogram of Moran's I random permutations against global Moran's I value (red bar), on *T. marmoratus'* final habitat suitability residuals (p-value = 0.078):

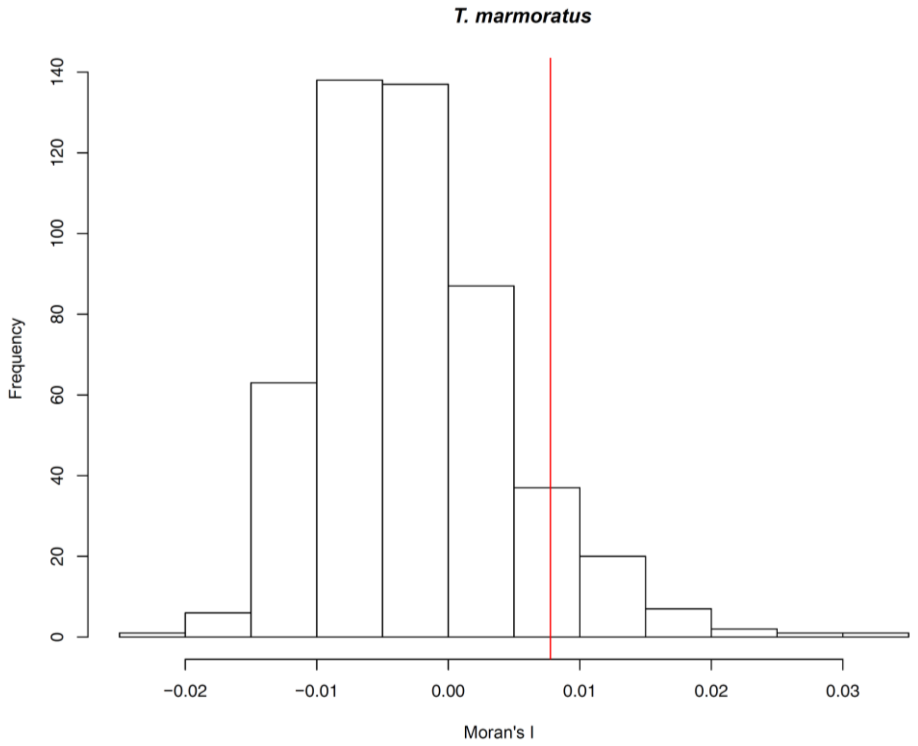

Supplement: Supplementary file 1 — Supplementary information [file 41598_2020_60479_MOESM1_ESM.pdf]
